# Mutational signatures and their association with survival and gene expression in urological carcinomas

**DOI:** 10.1016/j.neo.2023.100933

**Published:** 2023-09-06

**Authors:** Peeter Karihtala, Outi Kilpivaara, Katja Porvari

**Affiliations:** a Department of Oncology, Helsinki University Hospital Comprehensive Cancer Center and University of Helsinki, Helsinki FI-00290, Finland; b Applied Tumor Genomics Research Program, Faculty of Medicine, University of Helsinki, Helsinki FI-00014, Finland; c Department of Medical and Clinical Genetics, Medicum, Faculty of Medicine, University of Helsinki, Helsinki, Finland; d HUSLAB Laboratory of Genetics, HUS Diagnostic Center, Helsinki University Hospital, Helsinki FI-00014, Finland; e Department of Pathology, Medical Research Center Oulu, Oulu University Hospital and University of Oulu, Oulu FI-90220, Finland

**Keywords:** Bladder carcinoma, COSMIC, Mutations, Renal cell carcinoma cancer, Prognosis, Signature, Urothelial cancer

## Abstract

Different sources of mutagenesis cause consistently identifiable patterns of mutations and mutational signatures that mirror the various carcinogenetic processes. We used publicly available data from the Cancer Genome Atlas to evaluate the associations between the activity of the mutational signatures and various survival endpoints in six types of urological cancers after adjusting for established prognostic factors. The predictive power of the signatures was evaluated with dynamic area under curve models. In addition, links between mutational signature activities and differences in gene expression patterns were analysed. APOBEC-related signature SBS2 was associated with improved overall survival (OS) and disease-specific survival (DSS) in bladder carcinomas in the multivariate analysis, while clock-like signature SBS1 predicted shortened DSS and progression-free interval (PFI) in clear cell renal cell carcinomas (ccRCC). In papillary renal cell carcinomas (pRCC), SBS45 was a predictor of improved outcomes, and APOBEC-related SBS13 was a predictor of worse outcomes. Gene expression analyses revealed various enriched pathways between the low- and high-signature groups. Interestingly, in both the ccRCC and pRCC cohorts, the genes of several members of the melanoma antigen (MAGE) family were highly upregulated in the signatures, which predicted poor outcomes, and downregulated in signatures, which were associated with improved survival. To summarize, SBS signatures provide substantial prognostic value compared with just the traditional prognostic factors in certain cancer types. APOBEC-related SBS2 and SBS13 seem to provide robust prognostic information for particular urological cancers, maybe driven by the expression of specific groups of genes, including the MAGE gene family.

## Highlights

• Mutational signatures reflect carcinogenetic pathways.
• Several of mutational signatures provided relevant prognostic information.
• Gene expressions differed remarkably between the low- and high-signature groups.

## 1 Introduction

Urological cancers are among the most frequently occurring cancers, and they have a significant impact on total cancer mortality worldwide [1]. Their survival rates vary from the excellent long-term disease-specific survival (DSS) rates observed in testicular cancers and local prostate cancers to just a few years of median survival in cases of metastatic urinary bladder and renal cell carcinomas (RCC), despite the introduction of immuno-oncological treatments during the last few years [2,3]. Virtually all urological carcinomas lack any established prognostic factors in addition to the TNM stage and histological subtype. More precise and reproducible prognostic factors would thus be needed to optimize their surgical and oncological treatments, and surveillance.

Mutational signatures are characteristic patterns of mutations that reflect different carcinogenetic pathways [4]. The incidence of these unique mutational patterns in cancer genomes can give insights into, for example, past exposure to carcinogens and defects in DNA repair mechanisms. Single-base substitution (SBS) mutational signatures consist of 53 distinct signatures in the latest version of the Catalogue of Somatic Mutations in Cancer (COSMIC) database [5]. SBS signatures may tell, for example, about the activation of AID/APOBEC cytidine deaminases (signatures SBS2 and SBS13), defects in specific DNA proofreading mechanisms (e.g. SBS10), exposure to specific chemotherapies (e.g. SBS17), or they may be secondary to smoking (e.g. SBS4) [5,6]. The aetiology of some signatures is still unknown, although knowledge is rapidly progressing [5].

The association between COSMIC mutational signatures and outcomes in patients with urological cancers has not yet been studied. We used publicly available data from the Cancer Genome Atlas (TCGA) database to evaluate whether these signatures would be able to show differences in survival endpoints in bladder transitional cell carcinoma (BTCC), clear cell RCC (ccRCC), papillary RCC (pRCC), renal chromophobic carcinoma, prostate adenocarcinoma, and testicular germ cell tumours (TGCT). These studies were complemented by gene expression analysis to gain insight into the potential molecular mechanisms connected to mutational signatures and survival.

## 2 Materials and methods

### 2.1 Data

Mutational signature activity data published in [5] were accessed from The International Cancer Genome Consortium (ICGC) data portal: https://dcc.icgc.org/releases/PCAWG/mutational_signatures/Signatures_in_Samples/SP_Signatures_in_Samples. The baseline characteristics for the cohorts used in this study are shown in the Supplementary Tables 1-6. The data comprised whole-genome sequenced tumours from the Pan-Cancer Analysis of Whole-Genomes (PCAWG) consortium and whole-exome sequenced tumours from TCGA. Here we concentrated to TCGA data, where only SBS and ID signatures were available for the samples. Open-access RNA-seq harmonized (aligned to GRCh38 reference genome) gene expression data (HTSeq generated counts) from TCGA were downloaded from the Genomic Data Commons (GDC) portal [8] using the R package *TCGAbiolinks* v2.16.4 [9].

### 2.2 Univariate survival analysis

The association between mutational signatures and overall survival (OS) was first tested in a univariate approach utilizing the R packages *survival* (v3.2–7) [10] and *survminer* (v0.4.8) [11]. Only TCGA primary tumour samples and patients with available vital status and survival/follow-up time were included. The association between signature activity and OS in each cancer type was analysed according to the following criteria: at least twenty samples had both signature and survival data, there were at least five death events among the patients, and there were at least five samples with nonzero signature activity. The association with survival between low-activity and high-activity tumours was then tested using the log-rank test for the given signature. Low-activity tumours were defined as those with a median or lower activity of the signature within the cancer type, and high-activity tumours were defined as those with above-median activity. For the association between the gene expression of the MAGE family genes and OS, log2-transformed RSEM counts were used, and the subjects were dichotomized (high and low expression) based on the mean gene expression values. Only primary tumour samples were considered. For each analysis, a Kaplan–Meier curve was plotted using the function *ggsurvplot* from the *survminer* package.

### 2.3 Multivariate survival analysis

Multivariate survival analysis was performed for BTCC, ccRCC, pRCC, RCC, prostate adenocarcinoma, and TGCT using TCGA data and the *survival* and *survminer* packages. For each cancer type, a Cox proportional hazard regression with multiple variables was fitted, considering all signatures or selected MAGE expressions with nonzero activity in at least 5% of patients within the cancer type and selected clinical variables. For each cancer type, a separate regression model was fitted for each of the following endpoints: OS, DSS, PFI and DFI. The function *cox.zph* was used to test the proportional hazard assumptions and plot the Schoenfeld residuals for each variable and the combined model. The function *coxph* was used to run the Cox regression. Forest plots illustrating the hazard ratios of each variable were generated using the function *ggforest* from the package *survminer* v0.4.8 [11]. In cases where no uncensored patients (i.e. patients with a qualifying progression event) with a valid value of a clinical variable were available, these variables were either left out, or patients with particular values of that variable were removed. Cross-tables were produced using the R package *gtsummary* [12].

### 2.4 Time-dependent area under curve (AUC)

Time-dependent AUC was calculated for each Cox proportional hazard regression with at least one significant COSMIC mutational signature variable. For each model the incident/dynamic AUC was estimated as proposed by Song and Zhou [13] using the *survAUC* R package across the entire range of endpoint timepoints with one-year intervals. The summary AUC measure (integrated AUC; iAUC) reported is the integral of AUC on the entire time range.

### 2.5 *MAGE* gene expressions as predictors of overall survival

Open-access RNA-seq harmonized (aligned to GRCh38 reference genome) gene expression data (HTSeq generated counts) of the The Cancer Genome Atlas (TCGA) Kidney Renal Clear Cell Carcinoma: TCGA-KIRC (ccRCC) and Cervical Kidney renal papillary cell carcinoma: TCGA-KIRP (pRCC) were downloaded from the Genomic Data Commons (GDC) using R package TCGAbiolinks, v. 2.16.4. Gene expression data were quantile normalized using the *preprocessCore* R package. For ccRCC the following genes from the *MAGE* family were considered: *MAGEB2, MAGEC2, MAGEA4*, and *MAGEA6* while for pRCC *MAGEA3, MAGEA11, MAGEA12, MAGEC1*, and *MAGEC2*. Patients were dichotomized based on median of each *MAGE* gene expression with patients higher than median classified as High for *MAGE.*

### 2.6 Differential expression analysis

The HTseq count matrix was filtered to include only subjects with available mutational signature information. Additionally, data from only primary tumour samples were considered. Duplicated samples (different aliquots of the same patient) were also excluded. Samples (subjects) were dichotomized into low and high mutational signature groups based on the population median mutational signature activity. Finally, the count matrix was filtered to exclude genes with fewer than 10 reads in total across all samples. Data normalization and differential expression analysis were performed using the R package DESeq2 [14] by contrasting gene expression in the high versus the low group for each cancer type and mutational signature combination. Differentially expressed genes were tested on the null hypothesis that the log2-fold change between groups was equal to 0 (*lfc0*). Significant genes were identified as those with an adjusted p-value of < 0.05 and an absolute log2-fold change of > 1.

### 2.7 Gene ontology enrichment

Overrepresentation tests of differentially expressed genes in gene ontology, biological processes, molecular function, and cellular component terms were performed using the R package *clusterProfiler* [15,16]. Hypergeometric test p-values were corrected for multiple testing using the Benjamini–Hochberg multiple testing adjustment. Terms with an adjusted p-value of < 0.05 were considered significantly enriched. Enriched terms were further visualized using the R package *clusterProfiler*.

## 3 Results

### 3.1 COSMIC mutational signatures and survival

First, we studied whether specific mutational signatures were associated with survival endpoints (overall survival (OS), DSS, disease-free interval (DFI), or progression-free interval (PFI)) in the six studied cancer types from the TCGA cohort. We performed Cox proportional hazard analyses, which are reported in more detail in Table 1.

**Table 1:** Summary of Cox proportional hazard (CoxPH) analyses from TCGA data. The clinical variables used in the models included age, gender (if applicable), tissue, or organ of origin, and primary diagnosis. American Joint Committee on Cancer (AJCC) staging was also included, except for prostate adenocarcinoma, where this variable was replaced with two Gleason score groups: (1) a Gleason score of < 7 and 7 (3 + 4) and (2) a Gleason score of > 7 and 7 (4 + 3).

Cancer type	Endpoint	Signatures eligible for the analysis	Excluded variables / variable levels	Variables with rejected proportional hazards assumptions	Signatures with p-value < 0.05 from the CoxPH test
Bladder transitional cell carcinoma	OS	SBS1, SBS2, SBS3, SBS5, SBS13	AJCC pathologic: stage I samples, Primary diagnosis: Carcinoma, Papillary adenocarcinoma and Squamous cell carcinoma samples, Tissue: Bladder neck and Ureteric orifice samples		SBS2 (Improved survival)
	DSS	SBS1, SBS2, SBS3, SBS5, SBS13	Same as above		SBS2 (Improved survival)
	DFI	SBS1, SBS2, SBS3, SBS5, SBS13	Tissue or organ of origin and AJCC pathologic stage and signature SBS3 not included in the model	SBS2, SBS5, SBS13	
	PFI	SBS1, SBS2, SBS3, SBS5, SBS13	AJCC pathologic: stage I samples, Primary diagnosis: Carcinoma, Papillary adenocarcinoma and Squamous cell carcinoma samples, Tissue: Bladder neck and Ureteric orifice samples	SBS1	SBS2 (Improved survival)
Clear cell renal cell carcinoma	OS	SBS1, SBS5, SBS40, SBS45, SBS52	Tissue or organ of origin and Primary diagnosis not included in the model	Age	
	DSS	SBS1, SBS5, SBS40, SBS45, SBS52	Same as above	SBS40	SBS1 (Decreased survival)
	DFI	SBS1, SBS5, SBS40, SBS45, SBS52	Tissue or organ of origin, Primary diagnosis and SBS45 not included in the model, AJCC pathologic stage IV samples		
	PFI	SBS1, SBS5, SBS40, SBS45, SBS52	Tissue or organ of origin and Primary diagnosis not included in the model	SBS45	SBS1 (Decreased survival)
Renal papillary cell carcinoma	OS	SBS1, SBS2, SBS5, SBS13, SBS45	Same as above	AJCC pathologic stage, gender, SBS1	SBS45 (Improved survival)
	DSS	SBS1, SBS2, SBS5, SBS13, SBS45	Same as above	AJCC pathologic stage	SBS45 (Improved survival)
	DFI	SBS1, SBS2, SBS5, SBS13, SBS45	Tissue or organ of origin and Primary diagnosis not included in the model, AJCC pathologic stage IV samples	AJCC pathologic stage	SBS13 (Decreased survival)
	PFI	SBS1, SBS2, SBS5, SBS13, SBS45	Tissue or organ of origin and Primary diagnosis not included in the model	AJCC pathologic stage, SBS1, SBS5	SBS13 (Decreased survival)
Renal chromophobic carcinoma	OS	SBS1, SBS5, SBS17a, SBS46	Tissue or organ of origin and Primary diagnosis not included in the model, AJCC pathologic stage and signatures SBS5 and SBS46 also as model did not converge		
	DSS	SBS1, SBS5, SBS17a, SBS46	Tissue or organ of origin and Primary diagnosis not included in the model, AJCC pathologic stage and signatures SBS5, SBS17a and SBS46 also as model did not converge		
	DFI	SBS1, SBS5, SBS17a, SBS46	Tissue or organ of origin and Primary diagnosis not included in the model, AJCC pathologic stage and all signatures as model did not converge		
	PFI	SBS1, SBS5, SBS17a, SBS46	Tissue or organ of origin and Primary diagnosis not included in the model, AJCC pathologic stage and signatures SBS5, SBS17a and SBS46 also as model did not converge		
Prostate adenocarcinoma	OS	SBS1, SBS5, SBS10b, SBS15, SBS40, SBS45	Tissue or organ of origin not included; samples with primary diagnosis ‘Adenocarcinoma with mixed subtypes’ or ‘Mucinous adenocarcinoma’, signatures SBS10b and SBS15 due to inflated coefficients		SBS40, SBS45 (Decreased survival)
	DSS	SBS1, SBS5, SBS10b, SBS15, SBS40, SBS45	Tissue or organ of origin not included; samples with primary diagnosis ‘Adenocarcinoma with mixed subtypes’ or ‘Mucinous adenocarcinoma’, Gleason group and signatures SBS10b and SBS15 due to inflated coefficients		SBS5, SBS40 (Decreased survival)
	DFI	SBS1, SBS5, SBS10b, SBS15, SBS40, SBS45	Tissue or organ of origin not included; samples with primary diagnosis ‘Adenocarcinoma with mixed subtypes’ or ‘Mucinous adenocarcinoma’		
	PFI	SBS1, SBS5, SBS10b, SBS15, SBS40, SBS45	Same as above		
Testicular germ cell tumours	OS	SBS1, SBS2, SBS5, SBS7a, SBS13, SBS15, SBS19, SBS24, SBS42, SBS44	Tissue or organ of origin not included, ‘AJCC pathologic stage’ subtypes combined into 3 stages, ‘Primary diagnosis’ categories with few samples combined into ‘Other’ group, signatures SBS2, SBS7a, SBS13, SBS15, SBS19, SBS24 and SBS44 due to inflated coefficients		
	DSS	SBS1, SBS2, SBS5, SBS7a, SBS13, SBS15, SBS19, SBS24, SBS42, SBS44	Tissue or organ of origin not included, ‘AJCC pathologic stage’ and ‘Primary diagnosis’ and signatures SBS1, SBS2, SBS7a, SBS13, SBS15, SBS19, SBS24 and SBS44 due to inflated coefficients		
	DFI	SBS1, SBS2, SBS5, SBS7a, SBS13, SBS15, SBS19, SBS24, SBS42, SBS44	Tissue or organ of origin not included, ‘AJCC pathologic stage’ due to inflated coefficients	SBS44	
	PFI	SBS1, SBS2, SBS5, SBS7a, SBS13, SBS15, SBS19, SBS24, SBS42, SBS44		Primary diagnosis, SBS2	

OS = overall survival; DSS = disease-specific survival; DFI = disease-free interval; PFI = progression-free interval; SBS = single-base substitution.

**Fig. 1:**
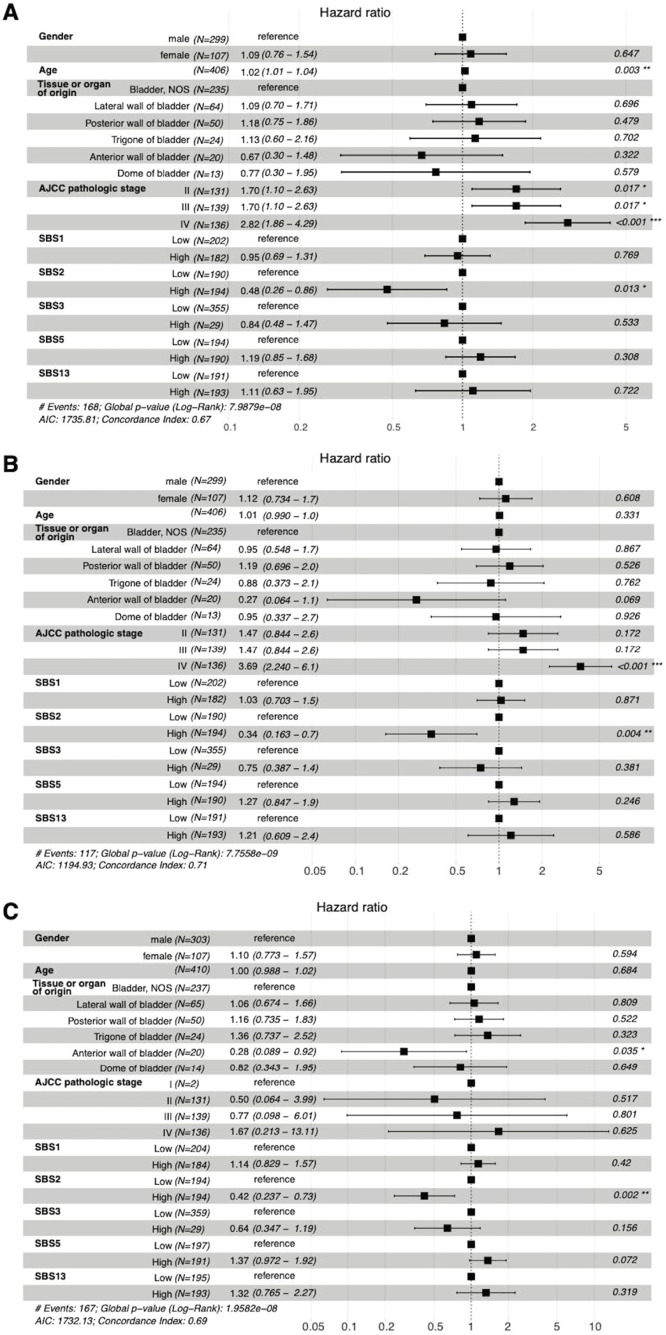
Cox proportional hazards regression analysis results for bladder transitional cell carcinoma. In these models, nonmetastatic disease and high SBS2 prevalence were associated with better OS (A) and DSS (B). A high number of SBS2 signatures also predicted a better PFI (C). AIC = Akaike Information Criterion; AJCC = American Joint Committee on Cancer; NOS = not otherwise specified; SBS = single-base substitution.

**Fig. 2:**
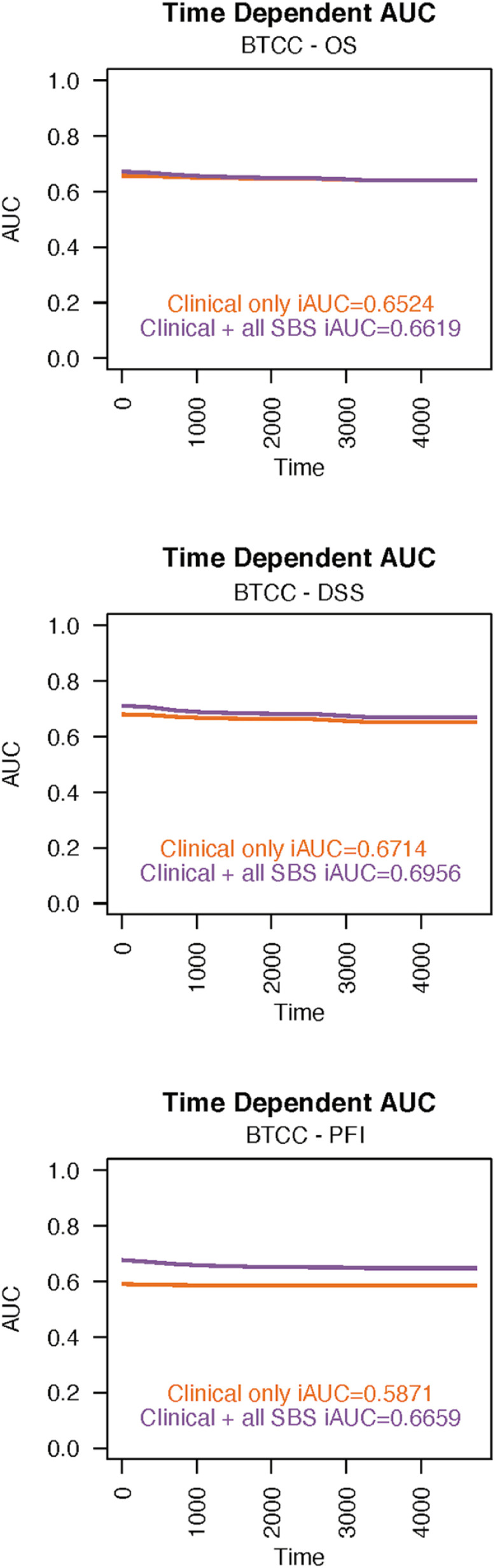
The time-dependent integrated areas under the curves (iAUC) values in bladder transitional cell carcinoma for progression-free interval (A), for disease-specific survival (B) and for overall survival (C). SBS = single-base substitution.

**Fig. 3:**
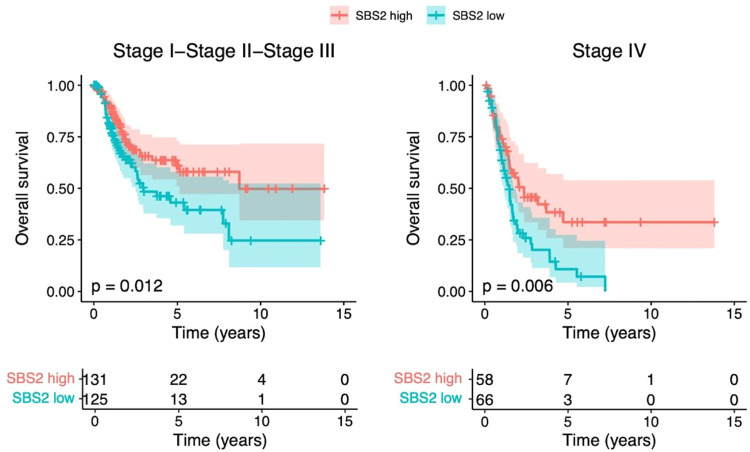
Kaplan–Meier curves showing that the prognostic value of SBS2 in terms of OS was observed separately in both stage I–III (left) and stage IV (right) bladder transitional cell carcinomas. The coloured areas represent 95% confidence intervals.

The high prevalence of SBS2 signatures in BTCC was associated in multivariate analysis with improved PFI (Hazard Ratio (HR) 0.42, 95% Confidence Interval (CI) 0.24–0.73), DSS (HR 0.34, 95% CI 0.16–0.70), and OS (HR 0.48, 95% CI 0.26–0.86; see Figure 1). The iAUC values in the Cox model were 0.6659 for PFI, 0.6956 for DSS and 0.6619 for OS (Figure 2.). Adding mutational signatures to the model improved especially the prognostic value of PFI when compared to using only the traditional prognostic factors. The prognostic value of the SBS2 signature also remained separate in the stage I–III and stage IV cohorts (Figure 3).

**Fig. 4:**
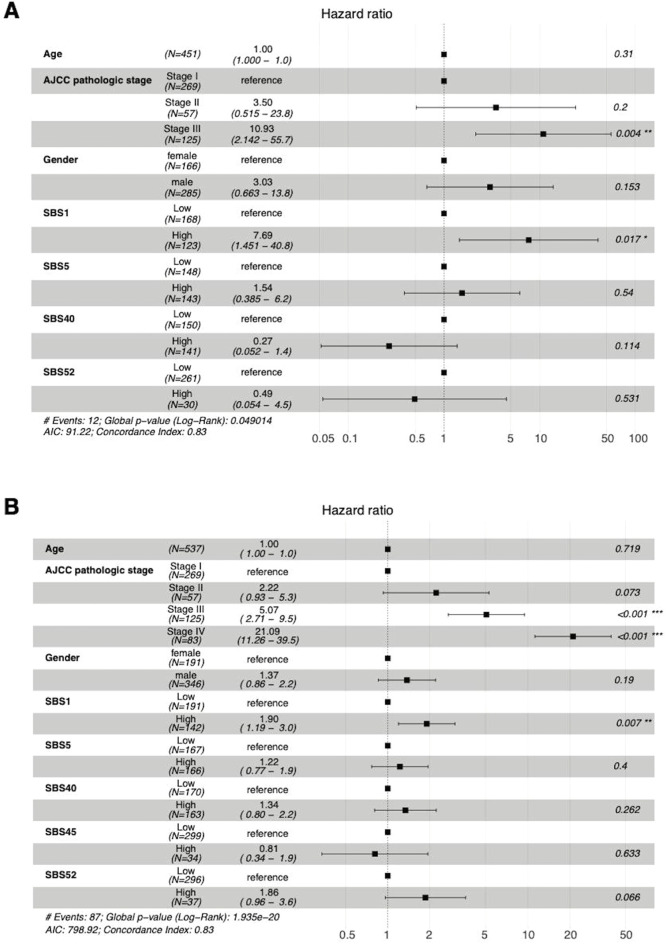
In patients with ccRCC, the high activity of SBS1 was associated with a worse DFI (A) and PFI (B). Stage was still the most predominant prognostic factor in both cases. AJCC = American Joint Committee on Cancer; SBS = single-base substitution.

**Fig. 5:**
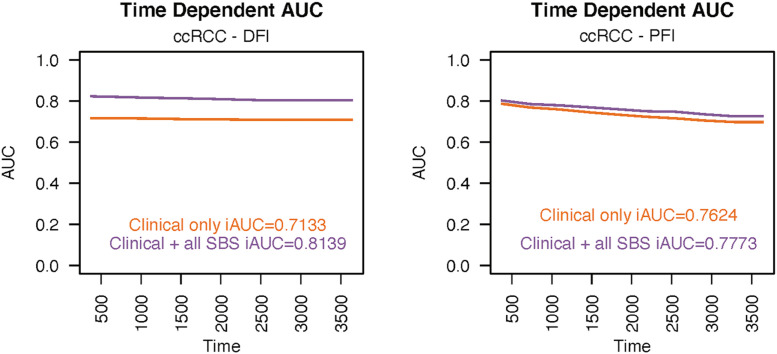
The time-dependent integrated areas under the curves show (iAUC) values in ccRCC for progression-free interval (A) and for disease-free interval (B). SBS = single-base substitution.

In ccRCC, the high prevalence of the SBS1 signature was associated with decreased DFI (HR 7.69, 95% CI 1.45–40.80) and PFI (HR 1.90, 95% CI 1.19–3.00; see Figure 4). The iAUC values were 0.8139 and 0.7773, respectively (Figure 5). There was a notable increase in the prognostic power of the DFI endpoint, when mutational signatures were added to the model apart from clinical prognostic factors.

**Fig. 6:**
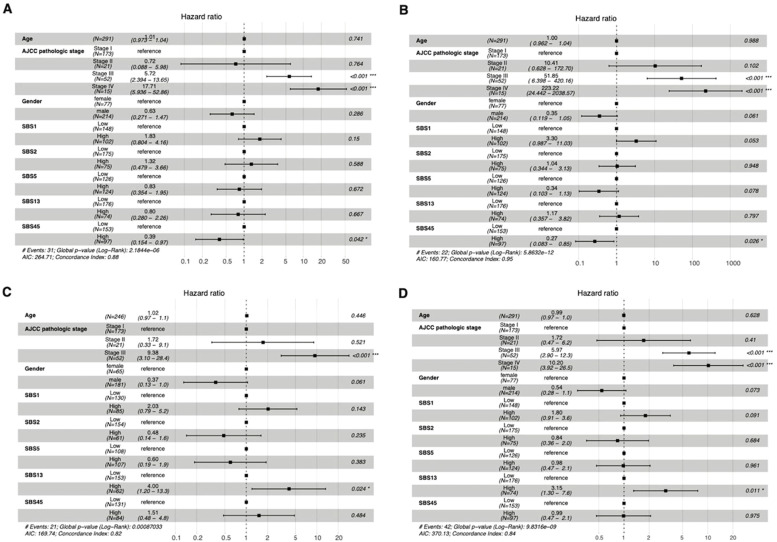
In pRCC patients, a high number of SBS45 signatures predicted longer OS (A) and DSS (B). Again, a high number of SBS13 signatures were associated with worse DFI (C) and PFI (D). AIC = Akaike information criterion; AJCC = American Joint Committee on Cancer; SBS = single-base substitution.

**Fig. 7:**
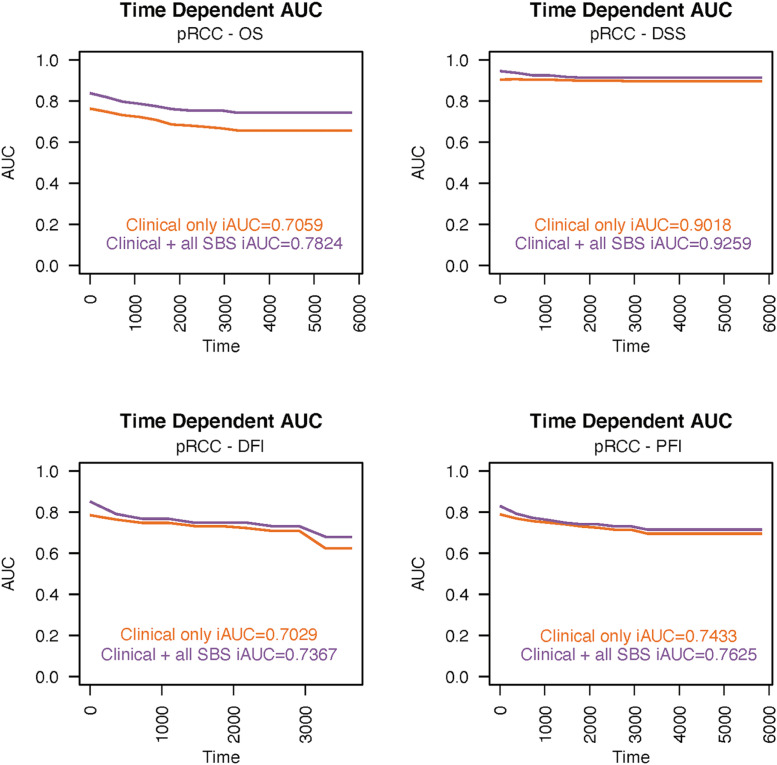
The integrated areas under the curves (iAUC) values in the pRCC cohort demonstrate the predictive power over time for disease-free interval (A), progression-free interval (B), disease-specific survival (C) and overall survival (D). SBS = single-base substitution.

Again, in pRCC, both SBS13 and SBS45 signatures were significantly associated with survival endpoints after adjusting for traditional prognostic factors (Figure 6). A high number of SBS13 signatures was associated with worse DFI (HR 4.00, 95% CI 1.20–13.30) and PFI (HR 3.15, 95% CI 1.30–7.60), while the presence of SBS45 was linked to improvements in the clinically more significant endpoints DSS (HR 0.27; 95% CI 0.083–0.85) and OS (HR 0.39; 95% CI 0.154–0.97). The predictive power analyzed for the Cox model with ROC showed iAUC value of 0.7367 for DFI, 0.7625 for PFI, 0.9259 for DSS and 0.7824 for OS, with substantial increase in the iAUC value especially with the OS endpoint (Figure 7).

**Fig. 8:**
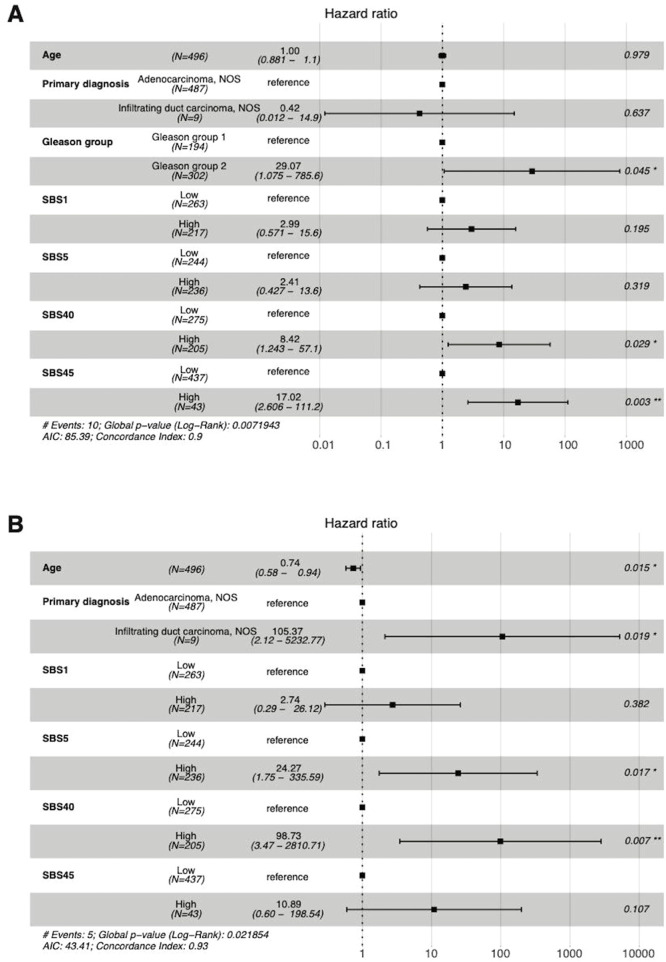
The high numbers of SBS40 and SBS45 signatures were predictors of poor OS (A) and SBS5 and SBS45 of DSS (B) in patients with prostate adenocarcinoma in Cox multivariate analysis. AIC = Akaike Information Criterion; AJCC = American Joint Committee on Cancer; NOS = not otherwise specified; SBS = single-base substitution.

**Fig. 9:**
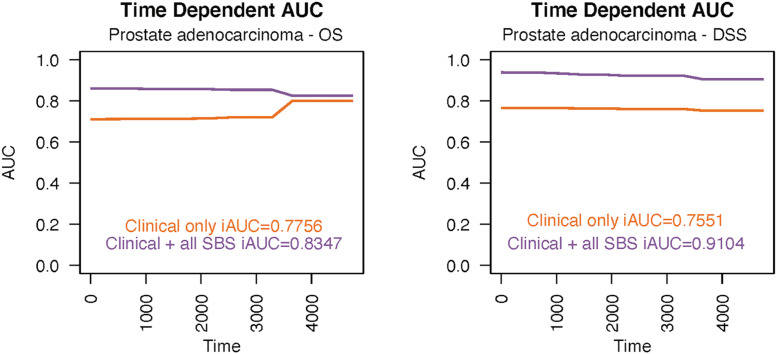
The integrated areas under the curves (iAUC) values in the prostate adenocarcinoma cohort demonstrate the predictive power over time for disease-specific survival (A) and overall survival (B). SBS = single-base substitution.

SBS5, SBS40, and SBS45 were found to be significant predictors of DSS and OS in prostate adenocarcinoma but with very wide CIs (Figure 8). Specifically, the high prevalence of SBS40 predicted both decreased DSS (HR 98.73; 95% CI 3.47–2810.71) and OS (HR 8.42; 95% CI 1.243–57.10). The SBS5 signature was associated with shorter DSS (HR 24.27; 95% CI 1.75–335.59) and SBS45 with shorter OS (HR 17.02; 95% CI 2.606–111.20). Adding mutational signatures to the traditional clinical prognostic factors yielded a lot more accurate predictive model, with all SBS iAUC values of 0.9104 for DSS and 0.8347 for OS (Figure 9).

No significant associations between survival and COSMIC mutational signatures were found in renal chromophobe carcinoma, or TGCT after adjusting for traditional prognostic factors.

### 3.2 Mutational signatures and gene expression analysis

Next, we identified putative links between mutational signature activity (low activity vs. high activity) and gene expression patterns based on TCGA data. To elucidate the potential drivers of the observed survival differences at the gene expression level, these analyses were conducted only for cancer types and signatures, in which statistically significant survival differences were observed in multivariate analyses.

Among the BTCC cohort, between SBS-low and SBS-high subjects, 550 genes were differentially expressed (absolute log2-fold change of > 1 and an adjusted p-value of < 0.05) (Supplementary Figure 1). The most downregulated gene in the SBS2-high group was delta-like 1 homologue, with 9.65-fold (95% CI 1.38; p = 1.9*10^−8^) expression in the SBS2-low group in patients with BTCC (Table 2). In the BTCC cohort, somatostatin was among the most downregulated genes in the SBS2 group.

**Table 2:** Examples of the differences in gene expression between the low- and high-signature cohorts. Renal chromophobic carcinoma and TCGT are not included in the table since there were no associations with survival outcomes in these cohorts in the multivariate analysis.

Cancer	Signature	Differentially expressed genes between low-signature and high-signature groups	Examples of differentially expressed genes between low-signature and high-signature groups (fold-change, adjusted p-value)	Examples of enriched GO terms
Bladder transitional cell carcinoma	SBS2 (association with increased survival)	550	Delta-like noncanonical Notch ligand 1 (9.65-fold downregulation, p = 1.9*10^−9^) Somatostatin (7.70-fold downregulation, p = 8.4*10^−8^)	Humoral immune response, regulation of metal ion transport
Clear cell renal cell carcinoma	SBS1 (association with decreased survival)	455	Melanoma antigen-B2 (10.1-fold upregulation, p = 1.8*10^−5^) Melanoma antigen-C2 (7.52-fold upregulation, p = 0.00078) Melanoma antigen A4 (6.82-fold upregulation, p = 0.019) Uromodulin (4.68-fold upregulation, p = 4.7*10^−5^) Melanoma antigen A6 (4.38-fold upregulation, p = 0.048)	Antigen binding, immunoglobulin genes, complement, T-cell/B-cell response related pathway upregulated
Papillary renal cell carcinoma	SBS13 (association with decreased survival)	842	Melanoma antigen-C2 (69.7-fold upregulation, p = 3.2*10^−8^) Melanoma antigen-C1 27.7-fold upregulation, p = 4.4*10^−5^) Melanoma antigen-A11 (12.2-fold upregulation, p = 0.013) Melanoma antigen-A3 (11.3-fold upregulation, p = 0.011) Melanoma antigen-A12 (10.3-fold upregulation, p = 0.000088) BUB1 (2.05-fold upregulation, p = 7.8*10^−7^) Survivin (2.2-fold upregulation, p = 3.1*10^−7^) Kallikrein-related peptidase 3 (9.45-fold downregulation, p = 7.9*10^−8^) Kallikrein 1 (7.69-fold downregulation, p = 1*10^−10^) Kallikrein-related peptidase 2 (5.36-fold downregulation, p = 0.00017)	Mitosis, cell division, nuclear segregation
Papillary renal cell carcinoma	SBS45 (association with increased survival)	337	Melanoma antigen-A3 (27.6-fold downregulation, p = 0.00011) Melanoma antigen-C2 (12.1-fold downregulation, p = 0.041) Melanoma antigen-C1 (7.98-fold downregulation, p = 0.042) Kallikrein-1 (5.00-fold upregulation, p = 3.9*10^−6^)	Several pathways related to ion channel activity downregulated
Prostate adenocarcinoma	SBS5 (association with decreased survival)	144	Cytochrome c oxidase subunit VIa polypeptide 1 (COX6A1) (3.03-fold downregulation, p = 0.002) Cytochrome c oxidase subunit 7B2 (5.23-fold upregulation, p = 0.01)	Related to vascular system
	SBS40 (association with decreased survival)	56	Prostate and testis expressed 1 (5.77-fold upregulation, p = 2.7*10^−10^)	Sperm function and spermatid development
	SBS45 (association with decreased survival)	108	Myosin light chain 3 (3.83-fold upregulation, p = 0.00034) Actin alpha 1, skeletal muscle (2.96-fold upregulation, p = 0.046) Troponin C1, slow skeletal, and cardiac type (2.62-fold upregulation, p = 0.016) Troponin I1, slow skeletal type 2.37-fold upregulation, p = 0.042)	Several pathways related skeletal muscles

GO = gene ontology; SBS = single-base substitution.

**Fig. 10:**
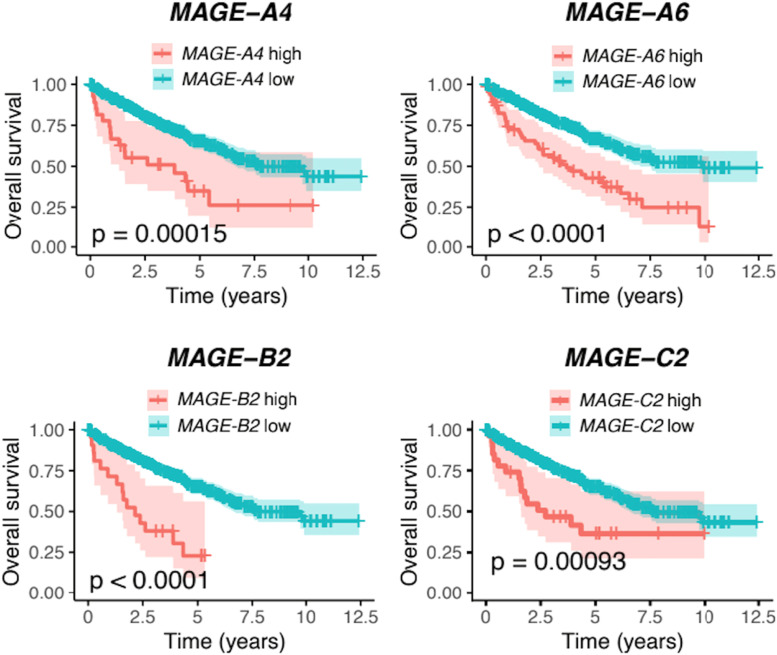
The high expressions of the *MAGE-B2, MAGE-C2, MAGE-A4,* and *MAGE-A6* genes were associated with worse overall survival in patients with clear cell renal cell carcinoma (ccRCC) in univariate analysis. These genes were significantly upregulated in patients with a high SBS1 signature (poor prognosis signature) in the ccRCC cohort.

**Fig. 11:**
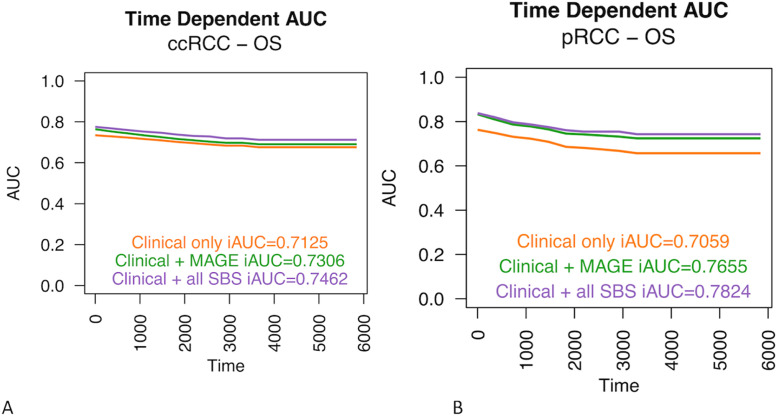
The expression of genes *MAGE-B2, MAGE-C2, MAGE-A4* or *MAGE-A6* provided only minor additional prognostic value over the traditional prognostic (clinical) values in the ccRCC cohort (A). In pRCC, the expression of *MAGE-A3*, *MAGE-A11*, *MAGE-A12*, *MAGE-C1*, and *MAGE-C2* genes was a better predictor when compared only to the traditional prognostic factors. This model also almost reached the prognostic level of mutational signatures and traditional prognostic factors combined (B).

In the ccRCC group, 455 genes were differentially expressed between SBS1-low and SBS1-high subjects. Of these genes, 343 were upregulated in the SBS1-high group, and 90 were immunoglobulin-related genes. The 22 most upregulated genes in the SBS1-high group included four members of the melanoma antigen (MAGE) family: *MAGE-B2* (10.1-fold expression, 95% CI 1.54, p = 0.000018), *MAGE-C2* (7.52-fold expression, 95% CI 1.56, p = 0.00078), *MAGE-A4* (6.81-fold expression, 95% CI 1.75, p = 0.018), and *MAGE-A6* (4.38-fold expression, 95% CI 1.64, p = 0.048). The high expressions of *MAGE-B2, MAGE-C2, MAGE-A4,* and *MAGE-A6* were all associated with poor OS (Figure 10). We also studied if *MAGE-B2, MAGE-C2, MAGE-A4* or *MAGE-A6* gene expression could provide prognostic power over the traditional prognostic factors in the ccRCC cohort, but the additional prognostic value of the gene expression was very limited (Figure 11A).

As another potentially interesting gene, encoding uromodulin (UMOD, Tamm–Horsfall protein), was 4.69-fold upregulated (95% CI 1.31, p = 4.7*10^−6^) in SBS1-high subjects in the ccRCC cohort. The five most enriched biological processes in SBS1-high patients were all related to immunoglobulins and immune responses (Supplementary Figure 2).

**Fig. 12:**
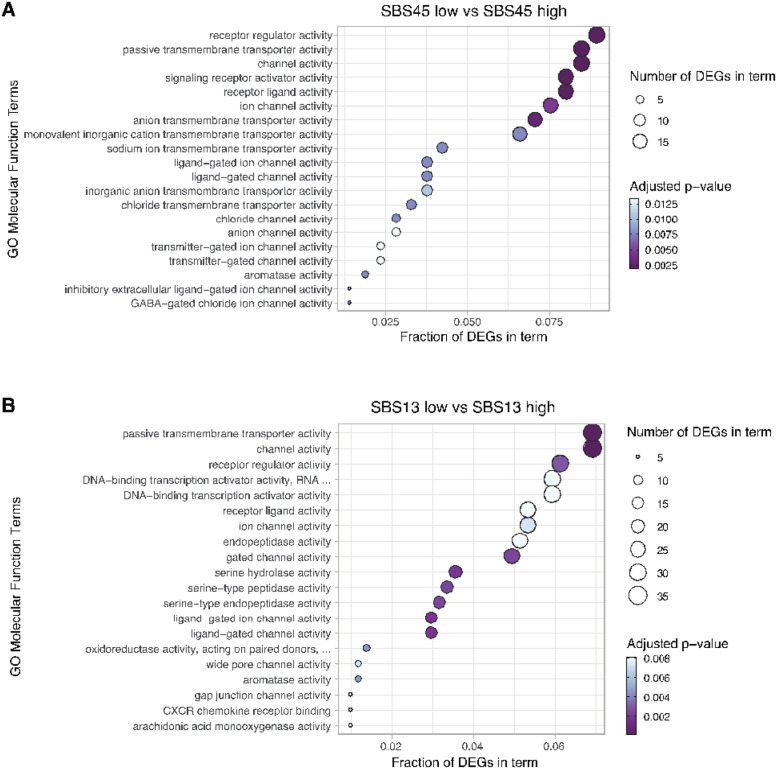
Top twenty significantly enriched (adjusted p-value of < 0.05) gene ontology molecular function terms in differentially expressed genes between SBS45-low and SBS45-high (A) and SBS13-low and SBS13-high (B) patients with papillary renal cell carcinoma.

High SBS45 levels, which were associated with improved DSS and OS in pRCC patients, were associated with 337 differentially expressed genes, of which 312 were downregulated in high SBS45-high patients compared to SBS45-low pRCC patients. *MAGE-A3* was the most downregulated gene in the SBS45-high group (27.6-fold change, 95% CI 1.90; p = 0.00011) in the pRCC cohort, and the 10 most downregulated genes also included MAGE family members *MAGEC2* and *MAGEC1*, with 12.1-fold change (95% CI 2.08, p = 0.041) and 7.98-fold (95% CI 1.84, p = 0.041) downregulations, respectively. Kallikrein-1 was the second most upregulated gene in the SBS45-high group in the pRCC cohort, with a 5.00-fold increase (95% CI 1.32, p = 3.9*10^−6^) compared to the SBS45-low group in the same cohort. The most enriched biological processes within the differentially expressed genes were associated predominantly with processes such as epidermis development, keratinocyte differentiation, and keratinization (Supplementary Figure 3). High SBS45 prevalence was also connected with several genes related to ion transport in patients with pRCC (Figure 12A).

**Fig. 13:**
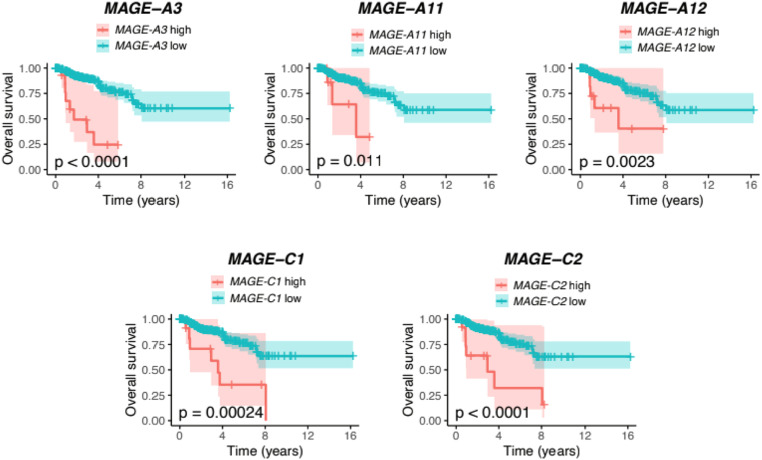
The high expressions of the *MAGE-A3, MAGE-A11, MAGE-*A12, MAGE*-C1,* and *MAGE-C2* genes were associated with worse overall survival in patients with papillary renal cell carcinoma (pRCC) in univariate analysis. These genes were significantly upregulated in patients with a high SBS13 signature (poor prognosis signature) in the pRCC cohort.

As described above, SBS13 was associated with worse outcomes for pRCC patients. Upon closer investigation of different gene expressions between high and low SBS13 phenotype individuals, 842 genes were differentially expressed between the SBS13 groups, and 744 of them were upregulated. For example, various enzyme activities belong to the most enriched gene ontology molecular function terms describing the differentially expressed genes between SBS13 low and high patient groups (Figure 12B). In contrast with the list of the most upregulated genes in the SBS45-high pRCC cohort, there was a significant upregulation of several MAGE family members in the SBS13-high pRCC cohort: *MAGE-C2* (69.7-fold upregulation, 95% CI 2.154, p = 5.1*10-6), *MAGE-C1* (27.7-fold upregulation, 95% CI 1.93, p = 4.4*10-5), *MAGE-A11* (12.2-fold upregulation, 95% CI 2.03, p = 0.013), *MAGE-A3* (11.3-fold upregulation, 95% CI 1.97, p = 0.011) and *MAGE-A12* (10.3-fold upregulation, 95% CI 1.70, p = 0.00088) in the SBS13-high group. The high expressions of *MAGE-A3, MAGE-C1,* and *MAGE-C2* were all associated with poor OS (Figure 13). When studied in the Cox model, the expression of *MAGE-A3*, *MAGE-A11*, *MAGE-A12*, *MAGE-C1*, and *MAGE-C2* genes was a better predictor than just the traditional prognostic factors, and this model almost reached the prognostic level of mutational signatures and traditional prognostic factors combined (Figure 11B). Again, kallikrein-related peptidase 3, kallikrein 1, and kallikrein-related peptidase 2 were among the most downregulated genes in the SBS13-high group. In particular, the processes linked to mitosis and nuclear segregation were upregulated in the patients with the worst prognoses, i.e. those with a high SBS13 prevalence (Supplementary Figure 4). Among those, BUB1 and survivin (BIRC5) upregulation were identified as genes of specific interest.

In patients with prostate adenocarcinoma, the SBS45 signature was associated with the enrichment of several pathways related to skeletal muscle development and regulation (Supplementary Figure 5). Again, between SBS40-high and SBS40-low patients, differentially expressed genes especially included those having functions in sperm and spermatid capacitation and function and in antibacterial and antimicrobial humoral responses (Supplementary Figure 6). Instead, SBS5 was associated with several regulators of blood pressure and vasoconstriction (Supplementary Figure 7). Mutational signatures were compared with clinical and pathological parameters, and these results are shown in Supplementary Tables 7–12.

### 3.3 Mutational burden

As expected, age-related mutational signatures SBS1 and SBS5, in particular, correlated with the total number of mutations (Supplementary Figure 8). The correlation between the individual signatures and the mutational burden was most frequently observed in the BTCC cohort, where SBS1, SBS2, SBS5, and SBS13 were associated with the total mutation count.

Mutation rate of MAGE family genes across urological cancers is shown in Supplementary Figure 9. Highest mutation rates were detected in the BTCC cohort, while mutations in MAGE genes were infrequent in other cohorts.

## 4 Discussion

This is the first study to assess the prognostic significance of COSMIC mutational signatures in urological cancers. One of the main findings was the association of a high number of SBS2 signatures with improved PFI, DSS, and OS in BTCC. In particular, PFI, and DSS have been considered reliable endpoints in this TCGA cohort [7]. The effect of SBS2 on DSS was of a similar magnitude as the traditionally with the most powerful predictor of outcome and stage in the multivariate analysis (HR 0.34, 95% CI 0.16–0.70). Notably, SBS2 activity was not associated with the traditional prognostic factors, and a survival benefit was observed in stage I–III and stage IV BTCCs. The SBS2 signature is formed due to APOBEC deamination of cytosine to uracil, and the APOBEC family of enzymes is a frequent source of hypermutation in most urothelial carcinomas [17]. While uracil subsequently enters the replication process uncorrected and pairs with A, this yields C-to-T mutations, which are characteristic of SBS2 [5,18,19]. Indeed, SBS2 is one of the most well-defined mutational signatures, and it is often accompanied by SBS13. Logically, with the hypermutator role of APOBEC, we observed a strict correlation between the number of SBS2 signatures and the total mutational burden in BTCC.

Recent data suggest that the APOBEC-driven subgroups of high SBS2 and SBS13 would form the largest subgroup of metastatic urothelial carcinomas and that SBS2 and SBS13 would be the major features of radiotherapy-associated BTCC [20,21]. APOBEC-related mutations also seem to drive the aggressiveness of non-muscle invasive BTCC and are associated with tobacco smoking, being an early, a critical event in bladder carcinogenesis [22], [23], [24], [25]. Patients with APOBEC-related mutations have doubled OS rates compared to patients with APOBEC-low BTCC [22]. The APOBEC-A-high SBS5 phenotype was linked to the absence of recurrences in a series of 62 high-grade, T1 non-muscle-invasive bladder cancers [26]. Non-COSMIC-based APOBEC mutational signatures have also been linked to improved outcomes in patients with advanced urothelial cancers, with most patients having BTCC [27]. To summarize, our results of a favourable prognosis in patients with a high number of SBS2 signatures are in line with the previously reported results regarding the behaviours of less aggressive cancers in APOBEC-high BTCC. Our results complete the previous reports by using COSMIC database, reporting the prognostic value from all stages, and using also additional endpoints than used in the previous studies. In gene expression analysis, we observed notable upregulation of, for example, delta-like noncanonical Notch ligand 1-gene expression in the SBS2-low group (worsened prognosis) in BTCC patients. This gene has been previously linked to a poor prognosis in a variety of different cancers [28], but has remained unstudied in bladder cancers *in vivo*.

In the ccRCC cohort, the high prevalence of the SBS1 signature provided additional prognostic information over staging in terms of decreased DFI and PFI. In particular, PFI has been considered a reliable endpoint in ccRCCs in TCGA data, with data containing 162 progression events in 362 evaluable patients [7]. SBS1 signatures are produced after the deamination of 5-methylcytosine to thymine, generating G:T mismatches in double-stranded DNA and further C-to-T substitutions [5]. The burden of SBS1 is associated with age in virtually all cancer types examined [29], but we still do not observe an association with OS in the ccRCC cohort.

The mutational load of ccRCCs is among the most common of all cancer types, and ccRCCs are consequently among the most immunologically active carcinomas. In our gene expression analysis, immune-related pathways were activated in SBS1-high (poor prognosis) subjects in the ccRCC cohort. Among these, 90 immunoglobulin genes were upregulated in patients with a high SBS1 signature. Several of these immunoglobulin genes have been associated with survival in ccRCC [30]. In line with our results, immune cell infiltration and enhanced immunological response are established adverse prognostic factors in ccRCC [31], [32], [33]. There is no previous literature on COSMIC signatures and prognosis in ccRCCs, and neither has the relationship between SBS1 and immune cell activation been previously evaluated in RCC. In primary and metastatic cutaneous melanomas, SBS1 activity was associated with lower activity of cytotoxic T and NK cells [34].

Several MAGE genes were upregulated in SBS1-high tumours in the ccRCC cohort. MAGE genes are highly conserved in all eukaryotes, and they play a crucial role in adaptation against environmental stress [35]. For example, *MAGE-B2,* which was the most upregulated gene in the SBS1-high ccRCC cohort, promotes stress tolerance, giving a growth advantage to cancer cells, and its high expression has been implicated in tumour growth and progression in various cancers *in vitro* but has not been studied previously in RCCs [35], [36], [37], [38]. *MAGE-C2*, with the observed 7.4-fold upregulation in SBS1 ccRCCs, is a cancer cell-specific regulator of TRIM28, and its high expression is associated with poor survival in various malignancies, including bladder and prostate carcinomas; however, the role of *MAGE-C2* has not yet been evaluated in RCCs [39], [40], [41], [42]. Other highly induced MAGE genes in the SBS1-high group in the ccRCC cohort included *MAGE-A4* and *MAGE-A6*. In particular, *MAGE-A6* seems to have significant pro-oncogenic roles [43,44], whereas the role of *MAGE-A4* is less well understood. The high RNA levels of *MAGE-B2, MAGE-A4,* and *MAGE-A6* were related to a dismal prognosis. A single potentially interesting gene, UMOD, was > 4.5-fold upregulated in SBS1-high subjects in the patients with ccRCC. For a small set of samples, UMOD RNA expression has been reported as one of the most upregulated transcripts in ccRCCs compared to benign renal tissue [45]. Based on our ccRCC results, it is possible that the dismal prognosis for SBS1-high patients may be driven by the upregulation of the immune system and MAGE family genes.

Of all renal cancers, pRCC accounts for approximately 15% and is genetically and morphologically distinct from other RCCs [46]. With the exception of staging, reliable prognostic factors are virtually absent in pRCC. According to our analyses, a high number of SBS13 signatures was associated with worse DFI and PFI in the TCGA pRCC cohort. SBS1 and SBS13 have also been attributed to the activity of AID/APOBEC family of cytidine deaminases [5,47]. However, while high APOBEC activity has been constantly linked to improved outcomes in BTCC, there are no previous data on the role of APOBEC in pRCC. In line with our results, high SBS13 activity is associated with a higher risk of postresection recurrence in non-small-cell lung cancers [48] and with radioiodine treatment resistance in papillary thyroid carcinomas [49]. We found that SBS13 activity was associated with the upregulation of 744 genes in pRCC, which were mainly linked to mitosis, cell division, and nuclear segregation. Notably, several MAGE family members were upregulated in pRCC in the SBS13 high cohort. The most upregulated of all genes in this cohort was *MAGE-C2*, which was upregulated 70-fold in the SBS13 high group in the pRCC cohort. In addition, *MAGE-C1, MAGE-A11, MAGE-A3,* and *MAGE-A12* were among the most upregulated genes, with at least a 10-fold increase in the SBS13 high group.

In urological cancers, *MAGE-A11* is best known for its protooncogenic role in prostate cancer [50]. Although MAGE family members in RCCs have rarely been studied, there is evidence that a specific single-nucleotide polymorphism of *MAGE-A11* provides prognostic information on OS in RCCs in materials of mixed histologies [51]. The *MAGE-A3* gene shares a 96% sequence similarity with more well-characterized prooncogenic *MAGE-A6* [52]. *MAGE-C1* is highly expressed in several cancers and has prognostic relevance in multiple myeloma and breast cancers [53,54]. The possible biological relationship between APOBEC enzymes and MAGE family members or kallikreins remains unstudied. However, it is known that the expression of *MAGE-A* genes is regulated by DNA methylation [55], which could be modified by APOBEC enzymes.

The high prevalence of SBS45 signatures was associated with improved OS and DSS in pRCCs, and in the time-dependent SBS45-including model the iAUC value was as high as 0.93 for DSS. Adding mutational signatures to the model with only traditional clinical variables increased especially the predictive power of OS analysis in the pRCC cohort. Among the SBS45-high patients, *MAGE-A3, MAGE-C1,* and *MAGE-C2* were among the most downregulated genes, and kallikrein 1 was highly upregulated in the SBS45-high patients. The function of these genes, and the respective proteins with tumour-suppressive functions, are well-characterised in prostate carcinogenesis, for example, but no previous literature exists on their role in RCCs [56,57]. The high RNA expression of *MAGE-A3, MAGE-C1*, and *MAGE-C2* individually associated with dismal survival rates in pRCC. According to the ROC analysis, gene expression of the five selected *MAGE* genes almost reached the prognostic significance of mutational signatures and traditional prognostic factors combined and exceeded substantially the prognostic power of that of just the traditional prognostic factors. In clinical practice, using gene expression analysis could be more feasible prognostic tool than mutational signatures requiring whole-exome sequencing (WES). It is worth noting that the SBS45 signature may be a sequencing artefact due to the use of 8-oxoguanine during sequencing [58].

In patients with prostate cancer, SBS5, SBS40, and SBS45 were associated with worse OS and DSS. There were very wide CIs in these analyses due to the limited number of events in this disease, which has a generally favourable prognosis. In addition, only PFI and DFI have been considered reliable endpoints in prostate cancer, while OS and DSS should be used with caution [7]. Thus, this study could not reveal any usable prognostic factors for use in patients with prostate cancer. We propose that the prognostic value of mutational signatures should be evaluated in the future, preferably in local high-risk or metastasized prostate cancers.

Despite urological TCGA cohorts containing a substantial number of patients with comprehensive clinical parameters, the major limitation of this study was the use of only a single dataset. WES is currently performed only to a minority of cancer patients, which limits the immediate implementation of the WES-based prognostic tools. As discussed above and by [7], the sample sizes in different TCGA cohorts vary considerably, and the usability of different survival endpoints is diverse. As TCGA contains only exome data, not all mutations in the human genome are covered. This is also a likely reason for our final analyses, including only SBSs, but not the other types of mutational signatures. Furthermore, mutations occurring at noncoding regulatory regions, including promoters, distal genomic enhancers, and silencer elements, might play crucial roles in gene expression levels. As the current study was not able to evaluate if the observed changes in gene expressions were drivers or passengers, this topic requires functional studies in the future. No significant associations between survival and COSMIC mutational signatures were found in chromophobe RCCs or in TGCT after adjusting for the traditional prognostic factors. This is due, at least partly, to the small number of events in both datasets.

## Conclusions

Several COSMIC signatures seem to provide prognostic information in urological cancers, and a significant proportion of the prognostic signatures are related to the activation of APOBEC enzymes. Based on the iAUC values, adding mutational signatures to Cox models seem to provide best prognostic information in pRCC with the iAUC values up to 0.93. The prognostic relevance was worst in BTCC, although mutational signatures are able to provide additional prognostic information also for BTCC patients over traditional prognostic factors. We presented a novel finding of the prominent upregulation of the genes belonging to the MAGE family in the groups where the signature predicted worse survival (SBS1 in ccRCC and SBS13 in pRCC) and the respective downregulation of MAGE genes in signature group with improved survival (SBS45 in pRCC). With using only selected MAGE genes we were also able to provide prognostic information near to that of mutational signatures and clinical variables combined. This suggests that MAGE genes could be key drivers of the current results and deserve further functional studies.

## Funding

No reportable funding was used to conduct this study.

## Author contributions

PK drafted the manuscript. All authors participated in the planning the study, evaluating the results and writing the final versions of the manuscript.

## Declaration of Competing Interest

The authors declare that they have no known competing financial interests or personal relationships that could have appeared to influence the work reported in this paper.

## Data Availability

The datasets used and analyzed are available on reasonable request.

## Appendix Supplementary materials

Image, application 1

Image, application 2

Image, application 3

Image, application 4

Image, application 5

Image, application 6

Image, application 7

Image, application 8

Image, application 9

Image, application 10

Image, application 11

Image, application 12
