# Supplementary material for: Mutational signatures and their association with survival and gene expression in urological carcinomas
Source: Neoplasia. 2023 Sep 6;44:100933. doi: 10.1016/j.neo.2023.100933 (PMC10495641; doi:10.1016/j.neo.2023.100933)
Supplement: Supplementary file 1 [file mmc1.docx]

| **Characteristic** | **N = 406**^1^ |
| --- | --- |
| Gender |  |
| male | 299 (74%) |
| female | 107 (26%) |
| Age | 69 (61, 77) |
| Tissue or organ of origin |  |
| Bladder, NOS | 235 (58%) |
| Lateral wall of bladder | 64 (16%) |
| Posterior wall of bladder | 50 (12%) |
| Trigone of bladder | 24 (5.9%) |
| Anterior wall of bladder | 20 (4.9%) |
| Dome of bladder | 13 (3.2%) |
| AJCC pathologic stage |  |
| II | 131 (32%) |
| III | 139 (34%) |
| IV | 136 (33%) |
| Pathologic T-class |  |
| T0 | 1 (0.3%) |
| T1 | 1 (0.3%) |
| T2 | 38 (10%) |
| T2a | 25 (6.7%) |
| T2b | 56 (15%) |
| T3 | 43 (11%) |
| T3a | 71 (19%) |
| T3b | 81 (22%) |
| T4 | 11 (2.9%) |
| T4a | 42 (11%) |
| T4b | 5 (1.3%) |
| Unknown | 32 |
| Pathologic N-class |  |
| N0 | 235 (59%) |
| N1 | 47 (12%) |
| N2 | 75 (19%) |
| N3 | 8 (2.0%) |
| NX | 35 (8.8%) |
| Unknown | 6 |
| Pathologic M-class |  |
| M0 | 192 (48%) |
| M1 | 11 (2.7%) |
| MX | 200 (50%) |
| Unknown | 3 |
| SBS1 |  |
| Low | 202 (53%) |
| High | 182 (47%) |
| Unknown | 22 |
| SBS2 |  |
| Low | 190 (49%) |
| High | 194 (51%) |
| Unknown | 22 |
| SBS3 |  |
| Low | 355 (92%) |
| High | 29 (7.6%) |
| Unknown | 22 |
| SBS5 |  |
| Low | 194 (51%) |
| High | 190 (49%) |
| Unknown | 22 |
| SBS13 |  |
| Low | 191 (50%) |
| High | 193 (50%) |
| Unknown | 22 |
| ^1^ n (%); Median (IQR) | |

Supplementary Table 1. Clinical and mutational signature summary statistics for patients in the Bladder translational cell carcinoma cohort. AJCC = American Joint Committee on Cancer; NOS = No otherwise specified; SBS = single-base substitution.
