# Supplementary material for: Mutational signatures and their association with survival and gene expression in urological carcinomas
Source: Neoplasia. 2023 Sep 6;44:100933. doi: 10.1016/j.neo.2023.100933 (PMC10495641; doi:10.1016/j.neo.2023.100933)
Supplement: Supplementary file 2 [file mmc2.docx]

| **Characteristic** | **N = 537**^1^ |
| --- | --- |
| Age | 61 (52, 70) |
| Unknown | 1 |
| AJCC pathologic stage |  |
| Stage I | 269 (50%) |
| Stage II | 57 (11%) |
| Stage III | 125 (23%) |
| Stage IV | 83 (16%) |
| Unknown | 3 |
| Gender |  |
| female | 191 (36%) |
| male | 346 (64%) |
| Pathologic T-class |  |
| T1 | 22 (4.1%) |
| T1a | 142 (26%) |
| T1b | 111 (21%) |
| T2 | 55 (10%) |
| T2a | 10 (1.9%) |
| T2b | 4 (0.7%) |
| T3 | 5 (0.9%) |
| T3a | 122 (23%) |
| T3b | 53 (9.9%) |
| T3c | 2 (0.4%) |
| T4 | 11 (2.0%) |
| Pathologic N-class |  |
| N0 | 240 (45%) |
| N1 | 17 (3.2%) |
| NX | 280 (52%) |
| Pathologic M-class |  |
| M0 | 426 (80%) |
| M1 | 79 (15%) |
| MX | 30 (5.6%) |
| Unknown | 2 |
| SBS1 |  |
| Low | 191 (57%) |
| High | 142 (43%) |
| Unknown | 204 |
| SBS5 |  |
| Low | 167 (50%) |
| High | 166 (50%) |
| Unknown | 204 |
| SBS40 |  |
| Low | 170 (51%) |
| High | 163 (49%) |
| Unknown | 204 |
| SBS45 |  |
| Low | 299 (90%) |
| High | 34 (10%) |
| Unknown | 204 |
| SBS52 |  |
| Low | 296 (89%) |
| High | 37 (11%) |
| Unknown | 204 |
| ^1^ Median (IQR); n (%) | |

Supplementary Table 2. Clinical and mutational signature summary statistics for patients in the clear cell renal cell carcinoma cohort. AJCC = American Joint Committee on Cancer; SBS = single-base substitution.
