# Supplementary material for: Mutational signatures and their association with survival and gene expression in urological carcinomas
Source: Neoplasia. 2023 Sep 6;44:100933. doi: 10.1016/j.neo.2023.100933 (PMC10495641; doi:10.1016/j.neo.2023.100933)
Supplement: Supplementary file 3 [file mmc3.docx]

| **Characteristic** | **N = 291**^1^ |
| --- | --- |
| Age | 62 (54, 71) |
| Unknown | 5 |
| AJCC pathologic stage |  |
| Stage I | 173 (66%) |
| Stage II | 21 (8.0%) |
| Stage III | 52 (20%) |
| Stage IV | 15 (5.7%) |
| Unknown | 30 |
| Gender |  |
| female | 77 (26%) |
| male | 214 (74%) |
| Pathologic T-class |  |
| T1 | 23 (7.9%) |
| T1a | 108 (37%) |
| T1b | 63 (22%) |
| T2 | 19 (6.5%) |
| T2a | 9 (3.1%) |
| T2b | 5 (1.7%) |
| T3 | 9 (3.1%) |
| T3a | 39 (13%) |
| T3b | 11 (3.8%) |
| T3c | 1 (0.3%) |
| T4 | 2 (0.7%) |
| TX | 2 (0.7%) |
| Pathologic N-class |  |
| N0 | 50 (17%) |
| N1 | 24 (8.3%) |
| N2 | 4 (1.4%) |
| NX | 212 (73%) |
| Unknown | 1 |
| Pathologic M-class |  |
| M0 | 95 (34%) |
| M1 | 9 (3.3%) |
| MX | 172 (62%) |
| Unknown | 15 |
| SBS1 |  |
| Low | 148 (59%) |
| High | 102 (41%) |
| Unknown | 41 |
| SBS2 |  |
| Low | 175 (70%) |
| High | 75 (30%) |
| Unknown | 41 |
| SBS5 |  |
| Low | 126 (50%) |
| High | 124 (50%) |
| Unknown | 41 |
| SBS13 |  |
| Low | 176 (70%) |
| High | 74 (30%) |
| Unknown | 41 |
| SBS45 |  |
| Low | 153 (61%) |
| High | 97 (39%) |
| Unknown | 41 |
| ^1^ Median (IQR); n (%) | |

Supplementary Table 3. Clinical and mutational signature summary statistics for patients in the papillary renal cell carcinoma cohort. AJCC = American Joint Committee on Cancer; SBS = single-base substitution.
