# Supplementary material for: Mutational signatures and their association with survival and gene expression in urological carcinomas
Source: Neoplasia. 2023 Sep 6;44:100933. doi: 10.1016/j.neo.2023.100933 (PMC10495641; doi:10.1016/j.neo.2023.100933)
Supplement: Supplementary file 4 [file mmc4.docx]

| **Characteristic** | **N = 496**^1^ |
| --- | --- |
| Age | 62 (57, 67) |
| Unknown | 10 |
| Primary diagnosis |  |
| Adenocarcinoma, NOS | 487 (98%) |
| Infiltrating duct carcinoma, NOS | 9 (1.8%) |
| Gleason group |  |
| Gleason score of >7 or 7 (3 + 4) | 194 (39%) |
| Gleason score of > 7 and 7 (4 + 3) | 302 (61%) |
| Pathologic T-class |  |
| T2a | 13 (2.7%) |
| T2b | 10 (2.0%) |
| T2c | 165 (34%) |
| T3a | 159 (33%) |
| T3b | 132 (27%) |
| T4 | 10 (2.0%) |
| Unknown | 7 |
| Pathologic N-class |  |
| N0 | 344 (81%) |
| N1 | 79 (19%) |
| Unknown | 73 |
| Clinical M-class |  |
| M0 | 454 (99%) |
| M1a | 1 (0.2%) |
| M1b | 1 (0.2%) |
| M1c | 1 (0.2%) |
| Unknown | 39 |
| SBS1 |  |
| Low | 263 (55%) |
| High | 217 (45%) |
| Unknown | 16 |
| SBS5 |  |
| Low | 244 (51%) |
| High | 236 (49%) |
| Unknown | 16 |
| SBS40 |  |
| Low | 275 (57%) |
| High | 205 (43%) |
| Unknown | 16 |
| SBS45 |  |
| Low | 437 (91%) |
| High | 43 (9.0%) |
| Unknown | 16 |
| ^1^ Median (IQR); n (%) | |

Supplementary Table 4. Clinical and mutational signature summary statistics for patients in the prostate adenocarcinoma cohort. Pathologic M-class was unavailable and thus the the clinical M-class is provided in this cohort. AJCC = American Joint Committee on Cancer; SBS = single-base substitution.
