# Supplementary material for: Mutational signatures and their association with survival and gene expression in urological carcinomas
Source: Neoplasia. 2023 Sep 6;44:100933. doi: 10.1016/j.neo.2023.100933 (PMC10495641; doi:10.1016/j.neo.2023.100933)
Supplement: Supplementary file 5 [file mmc5.docx]

| **Characteristic** | **N = 113**^1^ |
| --- | --- |
| Age | 51 (43, 60) |
| Gender |  |
| female | 51 (45%) |
| male | 62 (55%) |
| Pathologic T-class |  |
| T1 | 6 (5.3%) |
| T1a | 24 (21%) |
| T1b | 25 (22%) |
| T2 | 22 (19%) |
| T2a | 4 (3.5%) |
| T2b | 7 (6.2%) |
| T3 | 2 (1.8%) |
| T3a | 18 (16%) |
| T3b | 3 (2.7%) |
| T4 | 2 (1.8%) |
| Pathologic N-class |  |
| N0 | 48 (42%) |
| N1 | 4 (3.5%) |
| N2 | 2 (1.8%) |
| NX | 59 (52%) |
| Pathologic M-class |  |
| M0 | 34 (65%) |
| M1 | 2 (3.8%) |
| MX | 16 (31%) |
| Unknown | 61 |
| SBS1 |  |
| Low | 26 (52%) |
| High | 24 (48%) |
| Unknown | 63 |
| SBS17a |  |
| Low | 43 (86%) |
| High | 7 (14%) |
| Unknown | 63 |
| ^1^ Median (IQR); n (%) | |

Supplementary Table 5. Clinical and mutational signature summary statistics for patients in the renal chromophobic carcinoma. SBS = single-base substitution.
