# Supplementary material for: Mutational signatures and their association with survival and gene expression in urological carcinomas
Source: Neoplasia. 2023 Sep 6;44:100933. doi: 10.1016/j.neo.2023.100933 (PMC10495641; doi:10.1016/j.neo.2023.100933)
Supplement: Supplementary file 6 [file mmc6.docx]

| **Characteristic** | **N = 134**^1^ |
| --- | --- |
| Age | 32 (26, 38) |
| Primary diagnosis |  |
| Seminoma, NOS | 66 (49%) |
| Embryonal carcinoma, NOS | 26 (19%) |
| Mixed germ cell tumor | 28 (21%) |
| Other | 14 (10%) |
| AJCC pathologic stage |  |
| Stage I | 101 (80%) |
| Stage II | 12 (9.4%) |
| Stage III | 14 (11%) |
| Unknown | 7 |
| Pathologic T-class |  |
| T1 | 76 (57%) |
| T2 | 51 (38%) |
| T3 | 6 (4.5%) |
| TX | 1 (0.7%) |
| Pathologic N-class |  |
| N0 | 46 (37%) |
| N1 | 11 (8.9%) |
| N2 | 2 (1.6%) |
| NX | 65 (52%) |
| Unknown | 10 |
| Pathologic M-class |  |
| M0 | 115 (97%) |
| M1 | 2 (1.7%) |
| M1a | 1 (0.8%) |
| M1b | 1 (0.8%) |
| Unknown | 15 |
| SBS1 |  |
| Low | 67 (52%) |
| High | 61 (48%) |
| Unknown | 6 |
| SBS5 |  |
| Low | 66 (52%) |
| High | 62 (48%) |
| Unknown | 6 |
| SBS42 |  |
| Low | 114 (89%) |
| High | 14 (11%) |
| Unknown | 6 |
| ^1^ Median (IQR); n (%) | |

Supplementary Table 6. Clinical and mutational signature summary statistics for patients in the testicular germ cell tumours cohort. AJCC = American Joint Committee on Cancer; NOS = No otherwise specified; SBS = single-base substitution.
