# Supplementary material for: Mutational signatures and their association with survival and gene expression in urological carcinomas
Source: Neoplasia. 2023 Sep 6;44:100933. doi: 10.1016/j.neo.2023.100933 (PMC10495641; doi:10.1016/j.neo.2023.100933)
Supplement: Supplementary file 7 [file mmc7.docx]

SBS1 SBS2 SBS3 SBS5 SBS13

| **Variable** | **Low**, N = 200*^1^* | **High**, N = 181*^1^* | **p-value***^2^* |  | **Low**, N = 192*^1^* | **High**, N = 189*^1^* | **p-value***^2^* |  | **Low**, N = 353*^1^* | **High**, N = 28*^1^* | **p-value***^2^* |  | **Low**, N = 192*^1^* | **High**, N = 189*^1^* | **p-value***^2^* |  | **Low**, N = 193*^1^* | **High**, N = 188*^1^* | **p-value***^2^* |
| --- | --- | --- | --- | --- | --- | --- | --- | --- | --- | --- | --- | --- | --- | --- | --- | --- | --- | --- | --- |
| **Age** | 67 (60, 76) | 70 (62, 78) | 0.020 |  | 68 (60, 77) | 70 (61, 77) | 0.27 |  | 69 (61, 77) | 66 (60, 76) | 0.43 |  | 67 (60, 77) | 70 (62, 76) | 0.16 |  | 67 (60, 77) | 70 (62, 76) | 0.13 |
| **Gender** |  |  | 0.24 |  |  |  | 0.010 |  |  |  | 0.66 |  |  |  | 0.16 |  |  |  | 0.045 |
| *female* | 56 (28%) | 41 (23%) |  |  | 60 (31%) | 37 (20%) |  |  | 89 (25%) | 8 (29%) |  |  | 55 (29%) | 42 (22%) |  |  | 58 (30%) | 39 (21%) |  |
| *male* | 144 (72%) | 140 (77%) |  |  | 132 (69%) | 152 (80%) |  |  | 264 (75%) | 20 (71%) |  |  | 137 (71%) | 147 (78%) |  |  | 135 (70%) | 149 (79%) |  |
| **Primary diagnosis** |  |  | 0.68 |  |  |  | 0.49 |  |  |  | 0.063 |  |  |  | 0.27 |  |  |  | 0.89 |
| *Transitional cell carcinoma* | 165 (82%) | 153 (85%) |  |  | 163 (85%) | 155 (82%) |  |  | 291 (82%) | 27 (96%) |  |  | 156 (81%) | 162 (86%) |  |  | 162 (84%) | 156 (83%) |  |
| *Papillary transitional cell carcinoma* | 35 (18%) | 28 (15%) |  |  | 29 (15%) | 34 (18%) |  |  | 62 (18%) | 1 (3.6%) |  |  | 36 (19%) | 27 (14%) |  |  | 31 (16%) | 32 (17%) |  |
| **Tissue or organ of origin** |  |  | 0.39 |  |  |  | 0.87 |  |  |  | 0.70 |  |  |  | 0.13 |  |  |  | 0.59 |
| *Bladder, not otherwise specified* | 116 (58%) | 106 (59%) |  |  | 115 (60%) | 107 (57%) |  |  | 207 (59%) | 15 (54%) |  |  | 115 (60%) | 107 (57%) |  |  | 114 (59%) | 108 (57%) |  |
| *Lateral wall of bladder* | 38 (19%) | 23 (13%) |  |  | 31 (16%) | 30 (16%) |  |  | 56 (16%) | 5 (18%) |  |  | 36 (19%) | 25 (13%) |  |  | 32 (17%) | 29 (15%) |  |
| *Posterior wall of bladder* | 24 (12%) | 23 (13%) |  |  | 23 (12%) | 24 (13%) |  |  | 44 (12%) | 3 (11%) |  |  | 21 (11%) | 26 (14%) |  |  | 21 (11%) | 26 (14%) |  |
| *Trigone of bladder* | 8 (4.0%) | 14 (7.7%) |  |  | 9 (4.7%) | 13 (6.9%) |  |  | 20 (5.7%) | 2 (7.1%) |  |  | 10 (5.2%) | 12 (6.3%) |  |  | 10 (5.2%) | 12 (6.4%) |  |
| *Anterior wall of bladder* | 8 (4.0%) | 10 (5.5%) |  |  | 10 (5.2%) | 8 (4.2%) |  |  | 17 (4.8%) | 1 (3.6%) |  |  | 4 (2.1%) | 14 (7.4%) |  |  | 12 (6.2%) | 6 (3.2%) |  |
| *Dome of bladder* | 6 (3.0%) | 5 (2.8%) |  |  | 4 (2.1%) | 7 (3.7%) |  |  | 9 (2.5%) | 2 (7.1%) |  |  | 6 (3.1%) | 5 (2.6%) |  |  | 4 (2.1%) | 7 (3.7%) |  |
| **AJCC pathologic stage** |  |  | 0.55 |  |  |  | 0.52 |  |  |  | 0.024 |  |  |  | 0.81 |  |  |  | 0.73 |
| *Stage II* | 68 (34%) | 59 (33%) |  |  | 65 (34%) | 62 (33%) |  |  | 122 (35%) | 5 (18%) |  |  | 65 (34%) | 62 (33%) |  |  | 65 (34%) | 62 (33%) |  |
| *Stage III* | 63 (32%) | 66 (36%) |  |  | 60 (31%) | 69 (37%) |  |  | 122 (35%) | 7 (25%) |  |  | 62 (32%) | 67 (35%) |  |  | 62 (32%) | 67 (36%) |  |
| *Stage IV* | 69 (34%) | 56 (31%) |  |  | 67 (35%) | 58 (31%) |  |  | 109 (31%) | 16 (57%) |  |  | 65 (34%) | 60 (32%) |  |  | 66 (34%) | 59 (31%) |  |

*^1^* Median (IQR); n (%)

*^2^* Wilcoxon rank sum test; Fisher's exact test; Fisher's Exact Test for Count Data with simulated p-value (based on 2000 replicates)

Supplementary Table 1. The associations between the traditional prognostic factors and signature activity in bladder translational cell carcinoma (TCGA cohort). AJCC = American Joint Committee on Cancer.
