# Supplementary material for: Mutational signatures and their association with survival and gene expression in urological carcinomas
Source: Neoplasia. 2023 Sep 6;44:100933. doi: 10.1016/j.neo.2023.100933 (PMC10495641; doi:10.1016/j.neo.2023.100933)
Supplement: Supplementary file 8 [file mmc8.docx]

|  | SBS1 |  | SBS5 |  | SBS17a |  | SBS46 |  |
| --- | --- | --- | --- | --- | --- | --- | --- | --- |
| **Variable** | **Low**, N = 26*^1^* **High**, N = 24*^1^* **p-value***^2^* |  | **Low**, N = 25*^1^* **High**, N = 25*^1^* **p-value***^2^* |  | **Low**, N = 43*^1^* **High**, N = 7*^1^* **p-value***^2^* |  | **Low**, N = 44*^1^* **High**, N = 6*^1^* | **p-value***^2^* |
| **Age** | 47 (36, 57) 51 (46, 67) 0.063 |  | 45 (36, 51) 57 (47, 68) 0.003 |  | 51 (43, 62) 47 (39, 56) 0.56 |  | 51 (42, 61) 44 (42, 49) | 0.44 |
| **Gender** | >0.99 |  | 0.56 |  | 0.69 |  |  | 0.18 |
| *female* | 10 (38%) 9 (38%) |  | 11 (44%) 8 (32%) |  | 17 (40%) 2 (29%) |  | 15 (34%) 4 (67%) |  |
| *male* | 16 (62%) 15 (62%) |  | 14 (56%) 17 (68%) |  | 26 (60%) 5 (71%) |  | 29 (66%) 2 (33%) |  |
| **Primary diagnosis** |  |  |  |  |  |  |  |  |
| *Renal cell carcinoma, chromophobe type* | 26 (100%) 24 (100%) 25 (100%) | | 25 (100%) 43 (100%) | | 7 (100%) 44 (100%) | | 6 (100%) | |
| **Tissue or organ of origin** |  |  |  |  |  |  |  |  |
| *Kidney, not otherwise specified* | 26 (100%) 24 (100%) | 25 (100%) | 25 (100%) | 43 (100%) | 7 (100%) | 44 (100%) | 6 (100%) |  |
| **AJCC pathologic stage** | 0.54 |  | 0.24 |  | 0.30 |  |  | 0.47 |
| *Stage I* | 11 (42%) 6 (25%) | 11 (44%) | 6 (24%) | 14 (33%) | 3 (43%) | 15 (34%) | 2 (33%) |  |
| *Stage II* | 8 (31%) 11 (46%) | 9 (36%) | 10 (40%) | 16 (37%) | 3 (43%) | 15 (34%) | 4 (67%) |  |
| *Stage III* | 6 (23%) 5 (21%) | 5 (20%) | 6 (24%) | 11 (26%) | 0 (0%) | 11 (25%) | 0 (0%) |  |
| *Stage IV* | 1 (3.8%) 2 (8.3%) | 0 (0%) | 3 (12%) | 2 (4.7%) | 1 (14%) | 3 (6.8%) | 0 (0%) |  |

*^1^* Median (IQR); n (%)

*^2^* Wilcoxon rank sum exact test; Fisher's exact test; Fisher's Exact Test for Count Data with simulated p-value (based on 2000 replicates)

Supplementary Table 2. The associations between the traditional prognostic factors and signature activity in renal chromophobic carcinoma (TCGA cohort). AJCC = American Joint Committee on Cancer.
