# Supplementary material for: Mutational signatures and their association with survival and gene expression in urological carcinomas
Source: Neoplasia. 2023 Sep 6;44:100933. doi: 10.1016/j.neo.2023.100933 (PMC10495641; doi:10.1016/j.neo.2023.100933)
Supplement: Supplementary file 9 [file mmc9.docx]

|  | | | | | | | | |  |  | | | | | | | | | |
| --- | --- | --- | --- | --- | --- | --- | --- | --- | --- | --- | --- | --- | --- | --- | --- | --- | --- | --- | --- |
|  |  | SBS1 |  |  |  | SBS5 |  |  |  | SBS40 |  |  |  | SBS45 |  |  |  | SBS52 |  |
| **Variable** | **Low**, N = 191*^1^* | **High**, N = 142*^1^* | **p-value***^2^* |  | **Low**, N = 167*^1^* | **High**, N = 166*^1^* | **p-value***^2^* |  | **Low**, N = 170*^1^* | **High**, N = 163*^1^* | **p-value***^2^* |  | **Low**, N = 299*^1^* | **High**, N = 34*^1^* | **p-value***^2^* |  | **Low**, N = 296*^1^* | **High**, N = 37*^1^* | **p-value***^2^* |
| **Age** | 0.16 (0.14, 0.18) | 0.17 (0.15, 0.20) | 0.013 |  | 0.16 (0.14, 0.18) | 0.17 (0.15, 0.20) | <0.001 |  | 0.16 (0.13, 0.18) | 0.17 (0.15, 0.20) | <0.001 |  | 0.17 (0.14, 0.19) | 0.17 (0.15, 0.19) | 0.75 |  | 0.17 (0.14, 0.19) | 0.17 (0.13, 0.19) | 0.64 |
| **Gender** |  |  | 0.73 |  |  |  | 0.22 |  |  |  | 0.91 |  |  |  | 0.46 |  |  |  | 0.15 |
| *female* | 71 (37%) | 56 (39%) |  |  | 58 (35%) | 69 (42%) |  |  | 64 (38%) | 63 (39%) |  |  | 112 (37%) | 15 (44%) |  |  | 117 (40%) | 10 (27%) |  |
| *male* | 120 (63%) | 86 (61%) |  |  | 109 (65%) | 97 (58%) |  |  | 106 (62%) | 100 (61%) |  |  | 187 (63%) | 19 (56%) |  |  | 179 (60%) | 27 (73%) |  |
| **Primary diagnosis** |  |  | 0.78 |  |  |  | 0.79 |  |  |  | >0.99 |  |  |  | >0.99 |  |  |  | 0.17 |
| *Clear cell adenocarcinoma, NOS* | 184 (96%) | 136 (96%) |  |  | 161 (96%) | 159 (96%) |  |  | 163 (96%) | 157 (96%) |  |  | 287 (96%) | 33 (97%) |  |  | 286 (97%) | 34 (92%) |  |
| *Renal cell carcinoma, NOS* | 7 (3.7%) | 6 (4.2%) |  |  | 6 (3.6%) | 7 (4.2%) |  |  | 7 (4.1%) | 6 (3.7%) |  |  | 12 (4.0%) | 1 (2.9%) |  |  | 10 (3.4%) | 3 (8.1%) |  |
| **Tissue or organ of origin** |  |  |  |  |  |  |  |  |  |  |  |  |  |  |  |  |  |  |  |
| *Kidney, NOS* | 191 (100%) | 142 (100%) |  | 167 (100%) | | 166 (100%) |  | 170 (100%) | | 163 (100%) |  | 299 (100%) | | 34 (100%) |  | 296 (100%) | | 37 (100%) |  |
| **AJCC pathologic stage** |  |  | 0.14 |  | |  | 0.32 |  | |  | 0.033 |  | |  | 0.58 |  | |  | 0.59 |
| *Stage I* | 109 (58%) | 66 (46%) |  | 86 (51%) | | 89 (54%) |  | 102 (60%) | | 73 (45%) |  | 157 (53%) | | 18 (53%) |  | 156 (53%) | | 19 (53%) |  |
| *Stage II* | 16 (8.5%) | 21 (15%) |  | 24 (14%) | | 13 (7.9%) |  | 14 (8.3%) | | 23 (14%) |  | 31 (10%) | | 6 (18%) |  | 35 (12%) | | 2 (5.6%) |  |
| *Stage III* | 43 (23%) | 36 (25%) |  | 38 (23%) | | 41 (25%) |  | 34 (20%) | | 45 (28%) |  | 73 (25%) | | 6 (18%) |  | 70 (24%) | | 9 (25%) |  |
| *Stage IV* | 21 (11%) | 19 (13%) |  | 19 (11%) | | 21 (13%) |  | 19 (11%) | | 21 (13%) |  | 36 (12%) | | 4 (12%) |  | 34 (12%) | | 6 (17%) |  |
| *Unknown* | 2 | 0 |  | 0 | | 2 |  | 1 | | 1 |  | 2 | | 0 |  | 1 | | 1 |  |

*^1^* Median (IQR); n (%)

*^2^* Wilcoxon rank sum test; Fisher's exact test; Fisher's Exact Test for Count Data with simulated p-value (based on 2000 replicates)

Supplementary Table 3. The associations between the traditional prognostic factors and signature activity in clear cell renal cell carcinoma (TCGA cohort). AJCC = American Joint Committee on Cancer; NOS = Not otherwise specified.
