# Supplementary material for: Mutational signatures and their association with survival and gene expression in urological carcinomas
Source: Neoplasia. 2023 Sep 6;44:100933. doi: 10.1016/j.neo.2023.100933 (PMC10495641; doi:10.1016/j.neo.2023.100933)
Supplement: Supplementary file 10 [file mmc10.docx]

|  | | | | | | |  | |  | | | | | | | | | |
| --- | --- | --- | --- | --- | --- | --- | --- | --- | --- | --- | --- | --- | --- | --- | --- | --- | --- | --- |
|  |  | SBS1 |  |  |  | SBS2 |  | | SBS5 |  |  |  | SBS13 |  |  |  | SBS45 |  |
| **Variable** | **Low**, N = 148*^1^* | **High**, N = 102*^1^* | **p-value***^2^* |  | **Low**, N = 175*^1^* | **High**, N = 75*^1^* | **p-value***^2^* **Low**, N = 126*^1^* | | **High**, N = 124*^1^* | **p-value***^2^* |  | **Low**, N = 176*^1^* | **High**, N = 74*^1^* | **p-value***^2^* |  | **Low**, N = 153*^1^* | **High**, N = 97*^1^* | **p-value***^2^* |
| **Age** | 62 (54, 70) | 63 (55, 73) | 0.29 |  | 62 (54, 70) | 64 (56, 74) | 0.25 58 (50, 65) | | 67 (60, 74) | <0.001 |  | 63 (54, 71) | 61 (52, 73) | 0.83 |  | 62 (53, 71) | 63 (55, 72) | 0.76 |
| *Unknown* | 3 | 2 |  |  | 3 | 2 | 3 | | 2 |  |  | 3 | 2 |  |  | 1 | 4 |  |
| **Gender** |  |  | 0.47 |  |  |  | 0.76 | |  | 0.024 |  |  |  | 0.44 |  |  |  | 0.47 |
| *female* | 38 (26%) | 31 (30%) |  |  | 47 (27%) | 22 (29%) | 43 (34%) | | 26 (21%) |  |  | 46 (26%) | 23 (31%) |  |  | 45 (29%) | 24 (25%) |  |
| *male* | 110 (74%) | 71 (70%) |  |  | 128 (73%) | 53 (71%) | 83 (66%) | | 98 (79%) |  |  | 130 (74%) | 51 (69%) |  |  | 108 (71%) | 73 (75%) |  |
| **Primary diagnosis** |  |  |  |  |  |  |  | |  |  |  |  |  |  |  |  |  |  |
| *Papillary adenocarcinoma, NOS* | 148 (100%) | 102 (100%) | 175 (100%) | | | 75 (100%) | 126 (100%) | | 124 (100%) | 176 (100%) | | | 74 (100%) | 153 (100%) | | | 97 (100%) | |
| **Tissue or organ of origin** |  |  |  |  |  |  |  | |  |  |  |  |  |  |  |  |  |  |
| *Kidney, NOS* | 148 (100%) | 102 (100%) |  | 175 (100%) | | 75 (100%) |  | 126 (100%) | 124 (100%) |  | 176 (100%) | | 74 (100%) |  | 153 (100%) | | 97 (100%) |  |
| **AJCC pathologic stage** |  |  | 0.004 |  | |  | 0.59 |  |  | 0.62 |  | |  | 0.47 |  | |  | 0.93 |
| *Stage I* | 99 (74%) | 55 (59%) |  | 113 (70%) | | 41 (62%) |  | 77 (66%) | 77 (69%) |  | 114 (70%) | | 40 (61%) |  | 95 (68%) | | 59 (67%) |  |
| *Stage II* | 14 (10%) | 5 (5.3%) |  | 12 (7.4%) | | 7 (11%) |  | 9 (7.7%) | 10 (9.0%) |  | 12 (7.4%) | | 7 (11%) |  | 11 (7.9%) | | 8 (9.1%) |  |
| *Stage III* | 17 (13%) | 25 (27%) |  | 29 (18%) | | 13 (20%) |  | 22 (19%) | 20 (18%) |  | 27 (17%) | | 15 (23%) |  | 25 (18%) | | 17 (19%) |  |
| *Stage IV* | 4 (3.0%) | 9 (9.6%) |  | 8 (4.9%) | | 5 (7.6%) |  | 9 (7.7%) | 4 (3.6%) |  | 9 (5.6%) | | 4 (6.1%) |  | 9 (6.4%) | | 4 (4.5%) |  |
| *Unknown* | 14 | 8 |  | 13 | | 9 |  | 9 | 13 |  | 14 | | 8 |  | 13 | | 9 |  |

*^1^* Median (IQR); n (%)

*^2^* Wilcoxon rank sum test; Fisher's exact test; Fisher's Exact Test for Count Data with simulated p-value (based on 2000 replicates)

Supplementary Table 4. The associations between the traditional prognostic factors and signature activity in papillary renal cell carcinoma (TCGA cohort). AJCC = American Joint Committee on Cancer; NOS = Not otherwise specified.
