# Supplementary material for: Mutational signatures and their association with survival and gene expression in urological carcinomas
Source: Neoplasia. 2023 Sep 6;44:100933. doi: 10.1016/j.neo.2023.100933 (PMC10495641; doi:10.1016/j.neo.2023.100933)
Supplement: Supplementary file 11 [file mmc11.docx]

|  |  | SBS1 |  |  |  | SBS5 |  |  |  | SBS10b |  |  |  | SBS15 |  |  |  | SBS40 |  |  |  | SBS45 |  |
| --- | --- | --- | --- | --- | --- | --- | --- | --- | --- | --- | --- | --- | --- | --- | --- | --- | --- | --- | --- | --- | --- | --- | --- |
|  | **Low**, N = | **High**, N = | **p-** |  | **Low**, N = | **High**, N = | **p-** |  | **Low**, N = | **High**, N = | **p-** |  | **Low**, N = | **High**, N = | **p-** |  | **Low**, N = | **High**, N = | **p-** |  | **Low**, N = | **High**, N = |  |
| **Variable** | 263*^1^* | 217*^1^* | **value***^2^* |  | 244*^1^* | 236*^1^* | **value***^2^* |  | 449*^1^* | 31*^1^* | **value***^2^* |  | 441*^1^* | 39*^1^* | **value***^2^* |  | 275*^1^* | 205*^1^* | **value***^2^* |  | 437*^1^* | 43*^1^* | **p-value***^2^* |
| **Age** | 61 (56, 66) | 64 (58, 67) | 0.001 |  | 61 (56, 66) | 63 (57, 67) | 0.073 |  | 62 (57, 67) | 59 (56, 66) | 0.21 |  | 62 (57, 67) | 62 (59, 67) | 0.27 |  | 62 (56, 67) | 62 (58, 67) | 0.38 |  | 62 (57, 67) | 62 (57, 66) | 0.84 |
| *Unknown* | 7 | 1 |  |  | 4 | 4 |  |  | 7 | 1 |  |  | 6 | 2 |  |  | 7 | 1 |  |  | 7 | 1 |  |
| **Primary diagnosis** |  |  | 0.74 |  |  |  | >0.99 |  |  |  | 0.45 |  |  |  | >0.99 |  |  |  | 0.31 |  |  |  | 0.19 |
| *Adenocarcinoma, NOS* | 259 (98%) | 212 (98%) |  |  | 239 (98%) | 232 (98%) |  |  | 441 (98%) | 30 (97%) |  |  | 432 (98%) | 39 (100%) |  |  | 268 (97%) | 203 (99%) |  |  | 430 (98%) | 41 (95%) |  |
| *Infiltrating duct carcinoma, NOS* | 4 (1.5%) | 5 (2.3%) |  |  | 5 (2.0%) | 4 (1.7%) |  |  | 8 (1.8%) | 1 (3.2%) |  |  | 9 (2.0%) | 0 (0%) |  |  | 7 (2.5%) | 2 (1.0%) |  |  | 7 (1.6%) | 2 (4.7%) |  |
| **Tissue or organ of origin** |  |  |  |  |  |  |  |  |  |  |  |  |  |  |  |  |  |  |  |  |  |  |  |
| *Prostate gland* | 263 (100%) | 217 (100%) |  | 244 (100%) | | 236 (100%) |  | 449 (100%) | | 31 (100%) |  | 441 (100%) | | 39 (100%) |  | 275 (100%) | | 205 (100%) |  | 437 (100%) | | 43 (100%) |  |
| **Gleason group** |  |  | 0.002 |  | |  | 0.78 |  | |  | 0.85 |  | |  | 0.50 |  | |  | 0.008 |  | |  | 0.42 |
| *Gleason group 1* | 118 (45%) | 67 (31%) |  | 96 (39%) | | 89 (38%) |  | 174 (39%) | | 11 (35%) |  | 168 (38%) | | 17 (44%) |  | 120 (44%) | | 65 (32%) |  | 166 (38%) | | 19 (44%) |  |
| *Gleason group 2* | 145 (55%) | 150 (69%) |  | 148 (61%) | | 147 (62%) |  | 275 (61%) | | 20 (65%) |  | 273 (62%) | | 22 (56%) |  | 155 (56%) | | 140 (68%) |  | 271 (62%) | | 24 (56%) |  |

*^1^* Median (IQR); n (%)

*^2^* Wilcoxon rank sum test; Fisher's exact test

Supplementary Table 5. The associations between the traditional prognostic factors and signature activity in prostate adenocarcinoma (TCGA cohort). NOS = Not otherwise specified.
