# Supplementary material for: Mutational signatures and their association with survival and gene expression in urological carcinomas
Source: Neoplasia. 2023 Sep 6;44:100933. doi: 10.1016/j.neo.2023.100933 (PMC10495641; doi:10.1016/j.neo.2023.100933)
Supplement: Supplementary file 12 [file mmc12.docx]

SBS1 SBS2 SBS5 SBS7a SBS13 SBS15 SBS19 SBS24 SBS42 SBS44

# Variable

**Low**, N

= 67*^1^*

32 (27,

**High**, N

= 61*^1^*

31 (25,

# p- value*^2^*

**Low**, N

= 120*^1^*

31 (25,

**High**, N = 8*^1^*

35 (32,

# p- value*^2^*

**Low**, N

= 66*^1^*

31 (25,

**High**, N

= 62*^1^*

32 (27,

# p- value*^2^*

**Low**, N

= 121*^1^*

31 (26,

**High**, N = 7*^1^*

36 (28,

# p- value*^2^*

**Low**, N

= 120*^1^*

31 (25,

**High**, N = 8*^1^*

35

# p- value*^2^*

**Low**, N

= 115*^1^*

32 (26,

**High**, N

= 13*^1^*

28 (26,

# p- value*^2^*

**Low**, N

= 118*^1^*

32 (26,

**High**, N

= 10*^1^*

31 (25,

|  | 38) | 36) |  | 38) | 35) |  | 38) | 36) |  |
| --- | --- | --- | --- | --- | --- | --- | --- | --- | --- |
| >0.99 |  |  | 0.69 |  |  | 0.73 |  |  | 0.94 |
|  | 58 | 4 |  | 57 | 5 |  | 58 | 4 |  |
|  | (49%) | (44%) |  | (50%) | (36%) |  | (48%) | (57%) |  |
|  | 24 | 2 |  | 23 | 3 |  | 25 | 1 |  |
|  | (20%) | (22%) |  | (20%) | (21%) |  | (21%) | (14%) |  |
|  | 23 | 3 |  | 22 | 4 |  | 24 | 2 |  |
|  | (19%) | (33%) |  | (19%) | (29%) |  | (20%) | (29%) |  |

# p- value*^2^*

**Low**, N

= 119*^1^*

32 (26,

**High**, N = 9*^1^*

29

# p- value*^2^*

**Low**, N

= 114*^1^*

32 (26,

|  | 32 | 31 |  |
| --- | --- | --- | --- |
| 0.98 | (26, | (29, | 0.95 |

**High**, N = 14*^1^*

31 (25,

# p- value*^2^*

**Low**, N

= 121*^1^*

**High**, N = 7*^1^*

# p- value*^2^*

**Age**

39)

35) 0.28

38)

38) 0.16

37)

39) 0.49

38)

38) 0.59

37)

(32,

39)

0.11

38)

32) 0.24

38)

37) 0.92

(27,

0.62

# Primary diagnosis

| *Seminoma,* | 47 | 15 | 56 6 |
| --- | --- | --- | --- |
| *NOS* | (70%) | (25%) | (47%) (75%) |

| 38 | 24 | 58 | 4 | 54 8 |
| --- | --- | --- | --- | --- |
| (58%) | (39%) | (48%) | (57%) | (45%) (100%) |

<0.001 0.072 0.14 >0.99 0.063 0.26

0%)

| 52 | 10 | 57 5 (5 |
| --- | --- | --- |
| (45%) | (77%) | (48%) |

0%) 0%)

| *Embryonal*  *carcinoma, NOS* | 9 (13%) | 17  (28%) | 26 0 (0%)  (22%) |
| --- | --- | --- | --- |
| *Mixed germ* | 6 | 20 | 26 0 (0%) |

|  | 10 | 16 |  | 25 | 1 |  | 26 0 ( |
| --- | --- | --- | --- | --- | --- | --- | --- |
|  | (15%) | (26%) |  | (21%) | (14%) |  | (22%) |
|  | 13 | 13 |  | 25 | 1 |  | 26 0 ( |
|  | (20%) | (21%) |  | (21%) | (14%) |  | (22%) |

|  | 25 | 1 |  | 24 2 (2 |
| --- | --- | --- | --- | --- |
|  | (22%) | (7.7%) |  | (20%) |
|  | 25 | 1 |  | 24 2 (2 |
|  | (22%) | (7.7%) |  | (20%) |
|  | 13 | 1 |  | 13 1 (1 |

*cell tumor*

(9.0%)

(33%)

(22%)

0%)

0%)

*Other* 5

| (7.5%) | 9 (15%) |  | (10%) | (25%) |  | (7.6%) | 9 (15%) |  | (11%) | (14%) |  | (12%) | 0 (0%) |  | (11%) | (7.7%) |  | (11%) | 0%) |  | (12%) | 0 (0%) |  | (11%) | (14%) |  | (12%) | 0 (0%) |  |
| --- | --- | --- | --- | --- | --- | --- | --- | --- | --- | --- | --- | --- | --- | --- | --- | --- | --- | --- | --- | --- | --- | --- | --- | --- | --- | --- | --- | --- | --- |
| **Tissue or organ of origin** |  |  |  |  |  |  |  |  |  |  |  |  |  |  |  |  |  |  |  |  |  |  |  |  |  |  |  |  |  |
| *Testis, NOS* 67 | 61 |  | 120 | 8 |  | 66 | 62 |  | 121 | 7 |  | 120 | 8 |  | 115 | 13 |  | 118 | 10 |  | 119 | 9 |  | 114 | 14 |  | 121 | 7 |  |
| (100%) | (100%) |  | (100%) | (100%) |  | (100%) | (100%) |  | (100%) | (100%) |  | (100%) | (100%) |  | (100%) | (100%) |  | (100%) | (100%) |  | (100%) | (100%) |  | (100%) | (100%) |  | (100%) | (100%) |  |
| **AJCC**  **pathologic stage** |  | 0.056 |  |  | 0.66 |  |  | 0.44 |  |  | 0.21 |  |  | 0.81 |  |  | 0.48 |  |  | 0.40 |  |  | 0.49 |  |  | 0.56 |  |  | 0.42 |

12 2 5

13 1 14

14 12 2 14

7 (7

| *Stage I* 51 | 47 |  | 90 | 8 |  | 52 | 46 |  | 93 | 5 |  | 91 | 7 |  | 86 | 12 |  | 91 |
| --- | --- | --- | --- | --- | --- | --- | --- | --- | --- | --- | --- | --- | --- | --- | --- | --- | --- | --- |
| (82%) | (77%) |  | (78%) | (100%) |  | (84%) | (75%) |  | (80%) | (71%) |  | (78%) | (100%) |  | (78%) | (92%) |  | (81%) |
| *Stage II* 2  (3.2%) | 9 (15%) |  | 11  (9.6%) | 0 (0%) |  | 4  (6.5%) | 7 (11%) |  | 9 (7.8%) | 2  (29%) |  | 11  (9.5%) | 0 (0%) |  | 10  (9.1%) | 1  (7.7%) |  | 10  (8.8%) |

| 0%) 91 7 87 11 93 5 | | | | |
| --- | --- | --- | --- | --- |
|  | (79%) | (88%) |  | (79%) (85%) (80%) (71%) |
|  | 10 | 1 |  | 11 0 (0%) 10 1 |

5 14 6

14 14 14

1 (10%)

(8

| .7%) (12%) (10%) |  | (8.6%) | (14%) |
| --- | --- | --- | --- |
| 14 0 (0%) 12 | 2 | 13 | 1 |

12

| *Stage III* | 9 (15%) | (8.2%) | (12%) | 0 (0%) | (9.7%) | 8 (13%) | (12%) | 0 (0%) | (12%) | 0 (0%) | (13%) | 0 (0%) | (11%) | 2 (20%) | (12%) |  | (11%) | (15%) | (11%) | (14%) |
| --- | --- | --- | --- | --- | --- | --- | --- | --- | --- | --- | --- | --- | --- | --- | --- | --- | --- | --- | --- | --- |
| *Unknown* | 5 | 0 | 5 | 0 | 4 | 1 | 5 | 0 | 4 | 1 | 5 | 0 | 5 | 0 | 4 | 1 | 4 | 1 | 5 | 0 |

*^1^* Median (IQR); n (%)

*^2^* Wilcoxon rank sum test; Fisher's Exact Test for Count Data with simulated p-value (based on 2000 replicates)

Supplementary Table 6. The associations between the traditional prognostic factors and signature activity in testicular germ cell tumors (TCGA cohort). AJCC = American Joint Committee on Cancer; NOS = Not otherwise specified.
